# USP10 regulates B cell response to SARS-CoV-2 or HIV-1 nanoparticle vaccines through deubiquitinating AID

**DOI:** 10.1038/s41392-021-00858-z

**Published:** 2022-01-04

**Authors:** Yuewen Luo, Xiantao Zhang, Ran Chen, Rong Li, Yang Liu, Junsong Zhang, Qin liu, Meijun Si, Jun Liu, Bolin Wu, Xuemei Wang, Shijian Wu, Yiwen Zhang, Xu Zhang, Deyin Guo, Xin He, Ting Pan, Hui Zhang

**Affiliations:** 1 Institute of Human Virology, Key Laboratory of Tropical Disease Control of Ministry of Education, Guangdong Engineering Research Center for Antimicrobial Agent and Immunotechnology, Zhongshan School of Medicine, Sun Yat-sen University, Guangzhou, 510080 China; 2 School of Medicine, Sun Yat-sen University, Guangzhou/Shenzhen, 510080 China; 3 Sun Yat-sen University Cancer Center, State Key Laboratory of Oncology in South China, Guangzhou, China; 4 Guangdong Provincial People’s Hospital, Guangdong Academy of Medical Sciences, Guangzhou, Guangdong China; 5 Guanghua School of Stomatology, Hospital of Stomatology, Sun Yat-sen University, Guangdong Provincial Key Laboratory of Stomatology, Guangzhou, 510060 Guangdong China; 6 National Guangzhou Laboratory, Bio-Island, Guangzhou, Guangdong 510320 China

**Keywords:** Immunology, Molecular biology

## Abstract

Activation-induced cytidine deaminase (AID) initiates class-switch recombination and somatic hypermutation (SHM) in antibody genes. Protein expression and activity are tightly controlled by various mechanisms. However, it remains unknown whether a signal from the extracellular environment directly affects the AID activity in the nucleus where it works. Here, we demonstrated that a deubiquitinase USP10, which specifically stabilizes nuclear AID protein, can translocate into the nucleus after AKT-mediated phosphorylation at its T674 within the NLS domain. Interestingly, the signals from BCR and TLR1/2 synergistically promoted this phosphorylation. The deficiency of USP10 in B cells significantly decreased AID protein levels, subsequently reducing neutralizing antibody production after immunization with severe acute respiratory syndrome coronavirus 2 (SARS-CoV-2) or human immunodeficiency virus type 1 (HIV-1) nanoparticle vaccines. Collectively, we demonstrated that USP10 functions as an integrator for both BCR and TLR signals and directly regulates nuclear AID activity. Its manipulation could be used for the development of vaccines and adjuvants.

## Introduction

Facing the challenge of antigens including those derived from numerous pathogens, individuals need to produce billions of antibodies using limited genes. Antibody diversification is initially developed through V(D)J recombination in the Ig gene, which occurs in the early B cells in the bone marrow (BM). Somatic hypermutation (SHM) is the second developing process for antibody diversification and occurs in germinal centers. SHM induces numerous point mutations in the variable region of the Ig genes, which provides the driving force for the antibody selection for various antigens, leading to the generation of higher-affinity antibodies.^1^ In addition, individuals need different types of antibodies to perform different functions, such as IgA antibody for mucosal immunity or IgE antibody for antiparasitic immunity. To this end, class-switch recombination (CSR) is required for multi-class antibody development. CSR replaces an upstream IgH constant (CH) region (e.g., Cμ) with a downstream CH region (e.g., Cγ, Cα, or Cε) to develop IgG, IgA, and IgE antibodies, respectively.^2^ SHM and CSR require AID.^3–6^ AID mediated the occurrence of CSR or SHM at different times and in different spaces. CSR usually occurs during the initial T-B cell interaction prior to germinal center formation and rapidly declines as B cells differentiate into GC cells and SHM initiates.^7^ AID is a key factor in humoral immunity and is related to the immune response to vaccines. In human immunized influenza H1N1 vaccination, AID activity in B cells is closely correlated with polyclonal antibody affinity maturation.^8^ To increase the humoral immune response to the vaccine, it is reasonable to increase the proper expression or activity of AID during antigen immunization.

The expression and activity of AID are tightly regulated in B cells at multiple levels, including transcription, post-transcription, and post-translational levels. Its disorders may mediate gene translocation between *IgH* and oncogene *C-Myc*, leading to the generation of B cell lymphomas.^9,10^ At the level of transcriptional regulation, TNF receptor families such as CD40, BAFF-R, TACI, and BCMA can activate the downstream NF-κB signals, including classical p65 and non-classical p52 NF-kb signals, to activate the transcription of *Aicda*.^11–15^ The stimulation of BCR signaling alone did not increase the expression of AID, even though BCR plays an important role in B cell development and antibody affinity. However, BCR signaling can play a synergistic role with TLR4 or CD40 signaling to promote the transcription of *Aicda*.^16^

Four small RNAs, including miR-155, miR-181b, miR-361, and miR-93, regulate AID expression by binding to the 3′UTR region of AID mRNA.^17–21^ Further studies showed that miR-155 expression was low in resting B cells, but increased with the stimulation of lipopolysaccharide (LPS) and interleukin 4 (IL-4), suggesting that miR-155 acts as a brake to prevent AID overexpression.^18^ In contrast, miR-181b is highly expressed in resting B cells, but its expression gradually decreases upon stimulation with LPS and IL-4.^19^ Bcl-6 is a key factor in the genesis and development of germinal center B (GC B) cells. Bcl-6 can directly inhibit the expression of miR-155 and miR-361, leading to the increase of AID in GC B cells.^17^

Post-translational modification (PTM) of AID directly regulates its activity and protein concentration. AID is phosphorylated at multiple sites including S3, T27, S38, T140, and T184.^22–26^ Phosphorylation of S38 and T140 increases the activity of AID,^24,27^ whereas S3 phosphorylation reduces its activity.^26^ AID is a nucleocytoplasmic shuttling protein.^28^ Although it remains in the cytoplasm in a steady state, its function is restricted to the nucleus, where its protein levels and activities are tightly controlled. Rapid regulation of proteins by ubiquitin ligases or deubiquitinases plays an important role in the functional regulation of immune cells and sustains normal functions of germinal centers.^29–32^ As both CSR and SHM require rapid and accurate expression and regulation of AID to ensure a normal immune response, it is better to regulate the process by directly regulating the protein concentration of AID via the ubiquitin-proteasome system (UPS) rather than AID transcription.^7^ Recent studies have found that AID binds to HSP90, EF1A, and HSP40-DnaJa1 in the cytoplasm to inhibit its degradation.^31–34^ Once AID is imported actively or passively into the nucleus, it is quickly exported back to the cytoplasm by CRM1 or degraded by the REG-γ mediated ATP-ubiquitin-independent proteasome pathway in the nucleus.^33–40^ In our previous study, we found that CUL7 specifically mediated AID ubiquitination by forming an E3 complex with FBXW11 and subsequently influenced the nuclear AID protein levels and antibody class-switch.^41^ However, whether the abundance of nuclear AID is regulated by deubiquitinases remains to be determined.

Here, we demonstrated the deubiquitinase USP10 as an interaction partner of AID using mass spectrometry. Further studies indicated that the nucleocytoplasmic shuttling protein USP10 is an AID-specific deubiquitinase that stabilizes nuclear AID protein. Its translocation into the nucleus is regulated by phosphorylation, which is initiated by BCR and TLR1/2 co-stimulation. In vitro and in vivo studies have indicated that suppression of USP10 activity inhibits class-switch and SHM. In addition, we found that mice with USP10 knockout in B cells showed a low humoral immune response after immunization with SARS-CoV-2 RBD-ferritin nanoparticle or HIV-1 eOD-GT8 60mer vaccine, when compared with wild-type mice, indicating the critical role of USP10 in nanoparticle vaccine response. By exploring the regulatory mechanisms of USP10, we found that BCR and TLR1/2 could synergistically regulate USP10 nuclear translocation, which inhibited AID degradation in the nucleus in an AKT-dependent manner. Finally, we immunized C57BL/6 mice with the SARS-CoV-2 RBD nanoparticle vaccine using the TLR2 activator Pam_3_CSK_4_ as an adjuvant. The immunized mice showed a higher immune response to the nanoparticle vaccine and produced more neutralizing antibodies compared to alum, which was used as the only adjuvant. However, the mice with USP10 knockout in B cells did not show this effect. Collectively, these studies revealed a novel regulatory system for the protein abundance of nuclear AIDs.

## Results

### USP10 is a specific deubiquitinase of AID

**Fig. 1:**
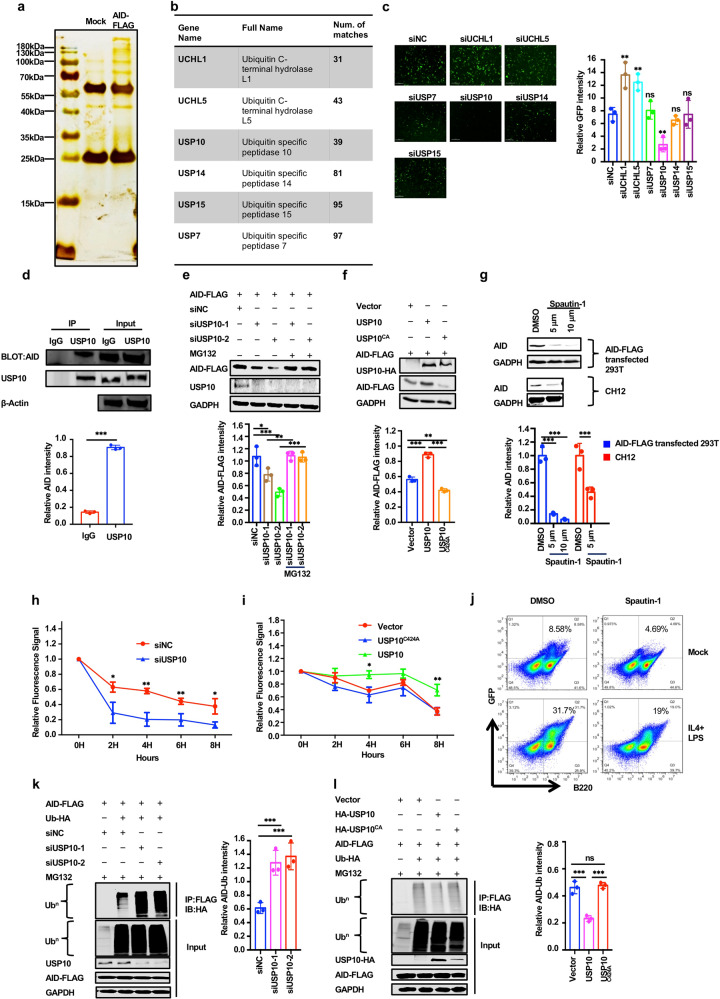
USP10 is a specific deubiquitinase of AID. **a** The 293 T cells were transfected with pcDNA3.1-AID-FLAG. At 48 h post-transfection, the cells were lysed for IP using anti-FLAG beads. AID-FLAG pull-down products were separated by SDS-PAGE gel and visualized by silver staining. **b** The deubiquitinating enzymes in identified AID-interacting proteins. The total peptide numbers of matches of each protein are indicated. **c** The fluorescence-field micrograph of identified AID-interacting deubiquitinase siRNA screen showed that USP10 knockdown dramatically decreased AID-GFP levels. Scale bar, 200 μm. **d** The internal Co-IP of USP10 and AID in CH12 cells. CH12 cells were stimulated with anti-CD40, IL-4, TGF-beta for 72 h to activate the expression of AID. The interaction of internal USP10 and AID was determined with IP and western blot assay. **e** The AID protein level was downregulated when USP10 was knocked down but rescued by MG132. The 293 T cells were co-transfected with AID-FLAG plasmid and control siRNA or siRNA specifically against USP10. The transfected cells were treated with MG132 for 24 h. The AID-FLAG levels and USP10 knockdown efficiency were determined by western blotting using anti-FLAG Abs and anti-USP10 Abs. GAPDH was used as a loading control. **f** The stabilization of AID by USP10 overexpression. The AID-FLAG expression plasmid DNA was co-transfected with USP10 or USP10^CA^ mutant into 293 T cells. The protein level of AID was confirmed by western blotting. GAPDH was used as a loading control. **g** Spautin-1 treatment promoted AID degradation. The 293 T cells were transfected with AID-FLAG plasmid, after 24 h, the transfected cells were treated with Spautin-1 in indicated concentrations (Top). The CH12 cells were stimulated with IL-4 (5 ng/ml), anti-CD40 (0.2 ug/ml), and TGF-β (5 ng/ml). After 48 h, the stimulated CH12 cells were treated with spatuin-1 in the indicated concentrations (Bottom). **h** USP10 knockdown promoted AID degradation. The 293 T cells were co-transfected with AID-Dendra2 plasmid and control siNC or siUSP10. After transfection 48 h, the transfected cells were exposed to a 405 nm light, so as to the Green fluorescence converse to Red fluorescence. The Red fluorescence signal was collected after expose 0, 2, 4, 6, or 8 h. The relative fluorescence signal was calculated through fluorescence intensity at a certain time divided by fluorescence intensity at 0 h. **i** The USP10 overexpression inhibited AID degradation. The 293 T cells were co-transfected with the plasmids expressing AID-Dendra2 and vector, with USP10 or USP10^CA^ mutant respectively. The AID-Dendra2’s decay was determined as described in (**h**). **j** Spautin-1 downregulated AID-GFP in spleen B cells of AID-GFP transgene mice. The spleen cells from AID-GFP transgene mice were stimulated with IL-4 (5 ng/ml) and LPS (25 μg/ml). After 48 h, the stimulated spleen cells were treated with Spatuin-1(5 μm) for 24 h. The AID-GFP protein level of B cells was determined by FACS. **k** USP10 knockdown promoted AID ubiquitination. The 293 T cells were transfected with siNC or siUSP10 and the indicated constructs were treated with MG132 for 12 h before harvest. AID-FLAG was immunoprecipitated with anti-FLAG beads and immunoblotted with anti-HA or anti-FLAG. GAPDH was used as a loading control. **l** USP10 overexpression decreased AID ubiquitination. The AID-FLAG plasmid and the indicated plasmid were co-transfected with USP10 or USP10^CA^ mutant into 293 T cells. The AID ubiquitination in transiently transfected cells was analyzed by CO-IP with anti-FLAG Abs and western blotting with anti-HA Abs. GAPDH as was used as a loading control. Data were representative of multiple experiments. *P < 0.05, **P < 0.01, ***P < 0.001

To identify the protein(s) affecting the activity of AID, we tried to identify the AID interacting proteins by expressing AID-FLAG in 293 T cells. The AID-interacting proteins were subject to co-immunoprecipitation with anti-FLAG-conjugated beads and then separated by SDS-PAGE. Multiple silver-stained bands, especially proteins over 100 kDa were observed (Fig. 1a). The visualized bands were excised and examined by mass spectrometry after trypsin digestion. In total 1194 proteins were identified. Among the interactors, 17 proteins were AID-specific interacting proteins (Supplementary Fig. 1c). We were especially interested in the factors involved in PTMs. According to Gene Ontology analysis, 61 proteins were involved in Ub-dependent catabolism, suggesting that ubiquitination regulation could be quite important for AID function (Supplementary Fig. 1a). Among the Ub-dependent catabolic-associated proteins, six were deubiquitinases (Fig. 1b and Supplementary Fig. 1b).

Because deubiquitinases can protect their interacting partners from UPS-mediated degradation, we speculated that AID protein levels could be maintained by a special deubiquitinase. To determine whether deubiquitinase was involved in AID stability, we used several siRNAs targeting these deubiquitinases that could putatively interact with AID for screening. We found that the AID protein level was significantly reduced when USP10 was knocked down (Fig. 1c). Furthermore, the specific interaction between internal USP10 and AID was confirmed in CH12 cells (Fig. 1d). Besides, the GST pull-down assay confirmed that AID directly bound with USP10 (Supplementary Fig. 1f). Therefore, we confirmed that USP10 is a specific interacting protein and a potential deubiquitinase for AID.

### USP10 knockdown or overexpression affects AID stability and ubiquitination

We further tested whether the stability and ubiquitination of AID were regulated by USP10. As shown in Fig. 1e, k, knockdown of USP10 disrupted AID stability and increased AID ubiquitination. In addition, proteasome inhibitor MG132 treatment prevented AID degradation even though USP10 was knocked down. Alternatively, the overexpression of USP10, but not its catalytically inactive CA mutant (C424A), stabilized AID and decreased the ubiquitination of AID (Fig. 1f, l).^42^ Dendra2 is a photoactivatable fluorescent protein that can be converted from green to red fluorescent states through exposure to 405 nm light and has been used to detect protein degradation.^43,44^ We generated an AID-Dendra2 fusion protein and measured the stability of AID-Dendra2 when USP10 was knocked down or overexpressed, according to our previously described method.^41^ The results showed that USP10 knockdown promoted AID-Dendra2 signal attenuation (Fig. 1h), while the overexpression of USP10 rather than USP10^CA^, delayed the attenuation of AID-Dendra2 (Fig. 1i). Additionally, after inhibition of USP10 activity with Spautin-1, a specific USP10 inhibitor, levels of both exogenous AID in 293 T cells and endogenous AID in CH12 cells decreased significantly (Fig. 1g).^45^ Meanwhile, an in vitro assay showed that spautin-1 treatment promoted AID-GFP degradation in spleen B220^+^ B cells isolated from AID-GFP transgene mice (Fig. 1j). These results suggest that USP10 is required for the deubiquitination and stability of AID.

### USP10 knockout CH12 cells showed a low protein level of AID and impaired the IgA class-switch

**Fig. 2:**
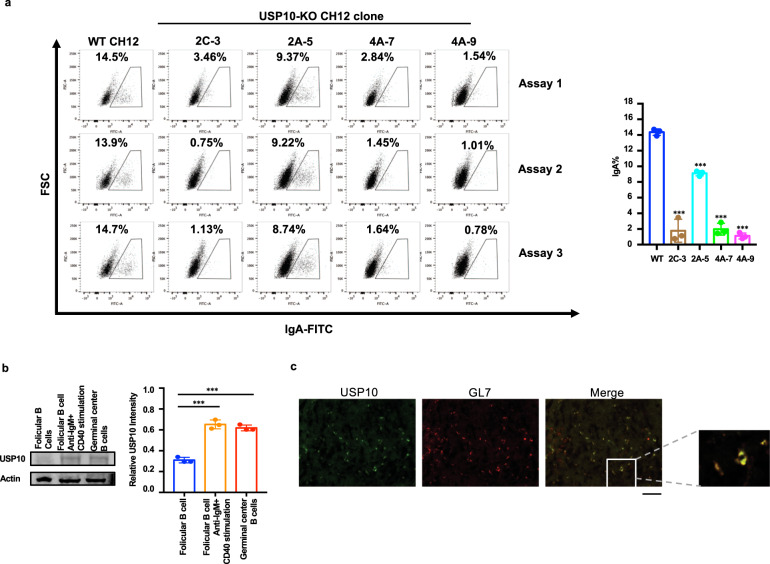
USP10-knocked out CH12 cells showed low levels of IgA class switching and USP10 was upregulated in germinal center B cells. **a** USP10 knockout CH12 cells showed low levels of IgA class switching. The USP10-knocked out CH12 cells of different single clone and the control NC clone were stimulated with IL-4, TGF-β, anti-CD40 according to the previously described concentration for 48 h. The IgA class-switch of CH12 cells was detected by FACS. **b** Western blot determined the USP10 expression in follicular B cells, anti-IgM-anti-CD40 stimulated follicular B cells, germinal center B cells. **c** USP10 expression in the GC B cells. GL7 was used for the stain of germinal center B cells. Data were representative of multiple experiments. *P < 0.05, **P < 0.01, ***P < 0.001

To functionally study the effect of USP10 on AID stability, we used the CRISPR-Cas9 technique to knockout *usp10* in CH12 cell lines (Supplementary Fig. 2a). The *usp10* gene in four single-clonal CH12 cell lines named 2C-3, 2A-5, 4A-7, and 4A-9 was successfully knocked out and validated by sequencing and western blotting (Supplementary Fig. 2b, c). These cell lines showed lower protein abundance of AID, higher ubiquitination level of AID, and impaired IgA class-switch compared with that in the control CH12 cell line after CIT (anti-CD40, IL-4, and TGF-β) stimulation (Fig. 2a and Supplementary Fig. 2d, e). As controls, the USP10 knockout cell lines exhibited the same α chain transcripts, μ chain transcripts in the *IgH* gene, and *Aicda* transcripts compared to the negative control (Supplementary Fig. 1g, h, i). These results indicate that USP10 regulates class-switch recombination directly by affecting AID protein abundance rather than AID transcription.

### USP10 is specifically expressed in GC B cells

To investigate USP10 expression in B cells, we initially examined the expression of USP10 using the public array database (GSE23925) and real-time RT-qPCR assay. The results showed that USP10 was upregulated in GC B or in vitro activated B cells (anti-IgM + CD40- stimulated B cells) compared with naïve follicular B (FoB) cells (Supplementary Fig. 1j, k). Western blotting further confirmed the enhancement of USP10 expression in either GC B cells or activated B cells (Fig. 2b). Furthermore, high USP10 expression in the GC B cells was confirmed by immunofluorescence staining (Fig. 2c).

### The mice with USP10 knockout in B cells exhibited a low abundance of AID, and impaired class-switch, somatic hypermutation, and affinity maturation

**Fig. 3:**
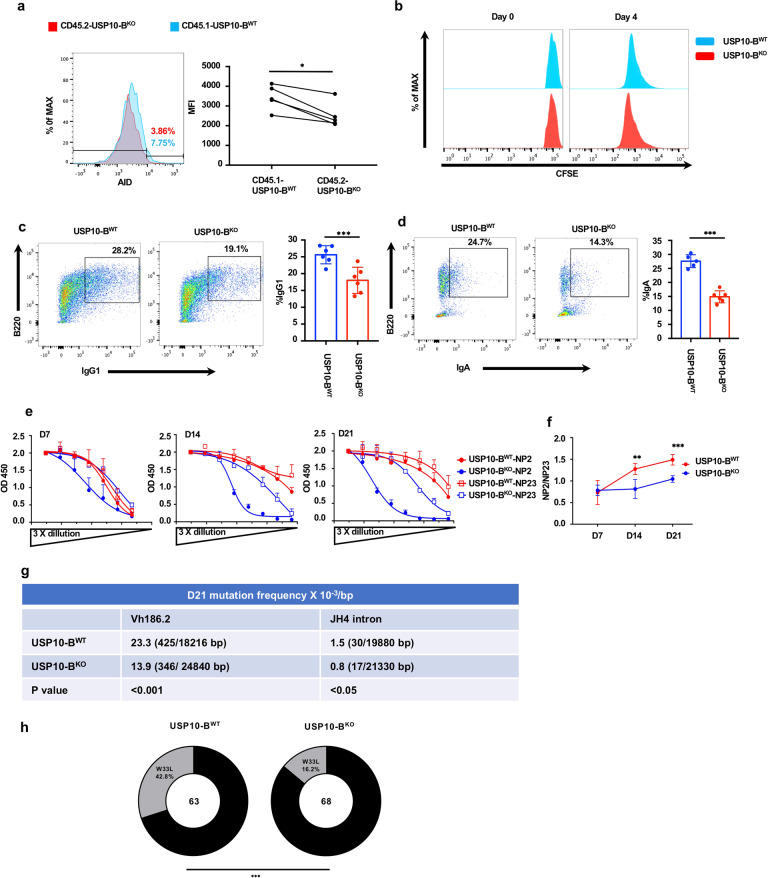
The mice with USP10 knockout in B cells exhibited a low abundance of AID, impaired class switching, somatic hypermutation, and affinity maturation. **a** Representative flow cytometry of AID protein levels in germinal center B cells (B220^+^GL7^+^CD95^+^) in 50:50 mixed bone marrow (BM) chimera mice using CD45.1-USP10-B^WT^and CD45.2-USP10-B^KO^ (*n* = 5). **b** Cell proliferation was assessed by staining cells with CFSE. **c**, **d** Representative flow cytometry of CSR to IgG1 (**C**), IgA (**D**) in splenic naïve B cells in USP10-B^WT^ or USP10-B^KO^ mice. **e–g** USP10-B^WT^ or USP10-B^KO^ mice were immunized with NP-CGG and analyzed at the time indicated. **e**, **f** NP2 and NP23 binding antibodies (total Ig) in sera of immunized mice were analyzed by ELISA. **e** OD values versus dilution factors are plotted. **f** Ratios of NP2/NP23 were calculated with raw OD value in a linear range. **g** The sorted GC B cells pooled from four mice of each genotype were used for the genomic DNA extraction and sequencing of the VH186.2 exon and JH4 intronic region. The total mutation frequencies are shown. The statistical analysis was performed with Fisher’s exact test. **h** The frequency of W33L mutation in the VH186.2 heavy chain was determined by sequencing. The total numbers of clones sequenced are indicated at the center of the pies. Statistical analysis was done with Fisher’s exact test. Data were representative of multiple experiments. *P < 0.05, **P < 0.01, ***P < 0.001

In order to investigate the effect of USP10 on B cells in mice, we commissioned Shanghai Model Organisms© to construct the USP10 conditional knockout mice through CRISPR-Cas9 technology. The mice were mated with CD19-cre mice to produce USP10-B^KO^ (*usp10* specifically knockout in B cells) Mice. The *usp10* gene knockout and validation strategies were shown in Supplementary Fig. 5a–g. To determine the AID stability when the absence of USP10 is intrinsic to B cells, we generated 50:50 mixed BM chimeric mice using CD45.1-USP10-B^WT^ and CD45.2-USP10-B^KO^ and analyzed the AID protein expression. As shown in Fig. 3A, CD45.2-USP10-B^KO^ exerted a lower level of AID protein compared to CD45.1-USP10-B^WT^ (Fig. 3a). To determine whether USP10 knockout in B cells influenced class-switch, CD43-negative naïve B cells in the spleen of the mice were sorted and stimulated with IL-4 and LPS for IgG1 class-switch and LPS, TGF-β, and anti-IgD for IgA class-switch. The results showed that both IgG1 and IgA class-switch were impaired in naïve B cells of USP10-B^KO^ mice (Fig. 3c, d). However, the USP10 knockout had no effect on IgG1 and IgA germline transcription (Supplementary Fig. 3a). In addition, USP10 knockout had no significant effect on B cell proliferation and apoptosis (Fig. 3b and Supplementary Fig. 3i). These results suggest that USP10 knockout in B cells decreases AID expression in B cells, thereby, decreasing IgG1 and IgA class-switch.

AID is required for SHM and antibody affinity maturation. To evaluate the effect of USP10 on AID-mediated SHM and affinity maturation, we first examined the BCR mutation frequency in NP-CGG-immunized USP10-B^WT^ and USP10-B^KO^ mice by antibody gene sequencing. After USP10-B^WT^ and USP10-B^KO^ mouse GC B cells were sorted by FACS, we sequenced the IgH VH186.2 germline, which encodes an NP-specific antibody, and the JH4 intronic region, which is irrelevant for affinity selection, thus, serves as a pure indicator of SHM efficiency. The overall mutation frequency of both JH4 and VH186.2 was substantially decreased in the absence of USP10 (Fig. 3g), suggesting that SHM was impaired. Notably, we found a significantly reduced fraction of the USP10-deficient GC B cell clone carrying the W33L mutation (Fig. 3h), which encodes a higher-affinity BCR for the NP antigen,^46^ indicating that USP10 deficiency could impair affinity maturation. To validate this notion, ELISA experiments were performed to measure NP binding antibody titers in the NP-CGG-immunized USP10-B^WT^ and USP10-B^KO^ mouse serum. The ratio of NP2 to NP23 binding antibody OD450 nm reads, which reflects anti-NP binding affinity, was markedly reduced in USP10-B^KO^ mice compared to that in USP10-B^WT^ mice (Fig. 3e, f). These results suggest that affinity maturation efficiency decreased in USP10-B^KO^ mice.

### USP10-B^KO^ mice exhibited a low immune response to HIV-1 and SARS-CoV-2 nanoparticle vaccines

The development of HIV-1 VRC01 broadly neutralizing antibodies (bnAbs) depends on the extremely high-frequency SHM of *IgH*.^47,48^ To further clarify whether USP10 knockout in B cells affects *IgH* SHM following the development of VRC01-type bnAbs, we used a VRC01 gH knock-in mouse that expresses a germline-reverted (unmutated ancestor) heavy chain of the broadly neutralizing HIV antibody VRC01. The mice developed VRC01-type bnAbs after immunization with HIV-1 eOD-GT8-60mer antigen.^49^ The eOD-GT8 nanoparticle vaccine contains a complete epitope of the CD4 binding site in gp120 of HIV-1 that strongly binds to the unmutated ancestor BCR of VRC01. We constructed the eOD-GT8-60mer vaccine, which displayed the eOD-GT8 on the surface of bacterial protein lumazine synthase (LS) to form 60 polymer nanoparticles and effectively activated VRC01-germline B cells (Supplementary Fig. 3b). The successful construction of the antigen was verified by staining with Coomassie blue and studying under an electron microscope (Supplementary Fig. 3c, d). USP10-BKO or USP10-BWT mice were immunized with the eOD-GT8-60mer antigen. VRC01-like antibody production was evaluated every 2 weeks by calculating the ratio of serum binding affinity to eOD-GT8 or eOD-GT8-KO [mutant (D368R, N279A, restore N276 glycosylation site) designed to block VRC01 binding] (Supplementary Figs. 3e–h, 4a). The results indicated that USP10 knockout affected the development of VRC01-type bnAbs. Taken together, these results revealed that USP10 plays a key role in AID-mediated CSR, SHM, and affinity maturation.

**Fig. 4:**
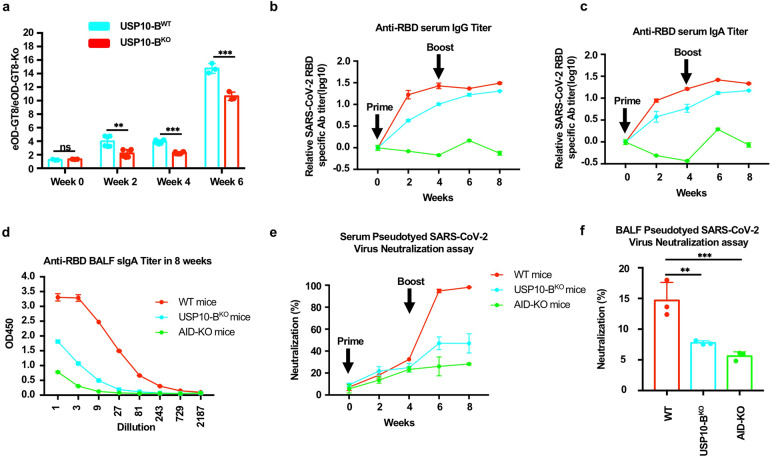
USP10-BKO mice exhibited a low immune response to SARS-CoV-2 RBD nanoparticle vaccines and HIV-1 eOD-GT8-60mer nanoparticle vaccines. **a** The ratios for the binding affinity of eOD-GT8 and eOD-GT8-KO were calculated at every 2 weeks. The eOD-GT8/eOD-GT8-KO ratio can reflect the development of VRC01 bnAbs. **b–f** The USP10-B^WT^, USP10-B^KO^, or AID-KO mice were prime/boost-vaccinated with 5 ug of SARS-CoV-2 RBD nanoparticle vaccine at week 0 and week 4. Sera were collected every 2 weeks. All the mice were euthanized at week 10. **b**, **c** The RBD-specific IgG, IgA relative titers each week was plotted as a time-course curve. **d** The bronchoalveolar lavage fluid (BALF) was collected from the euthanized mice in week 10. The RBD-specific sIgA titers were plotted as OD value versus dilution factors. **e** The neutralization of sera in the immunized mice was detected with the pseudotyped-SARS-CoV-2 infection (*n* = 3). The neutralizations at dilution of 1:100 of sera were shown. **f** The neutralization of BALF in immunized mice was detected with pseudotyped-SARS-CoV-2 infection (*n* = 3). The neutralizations at dilution of 1:100 of BALF were shown. Data were representative of multiple experiments. *P < 0.05, **P < 0.01, ***P < 0.001

Biomimetic nanoparticles are a good drug and antigen delivery system.^50^ Recently, our group designed a nanoparticle vaccine that covalently conjugates 24 copies of RBD protein subunits in the spike protein of the SARS-CoV-2 virus to the self-assembled *Helicobacter pylori* non-heme ferritin. The ferritin-based nanoparticle vaccine was able to induce abundant neutralizing antibodies in both mice and rhesus macaques.^51^ To evaluate the effect of USP10 on the immune response of SARS-CoV-2 RBD nanoparticles, USP10-B^WT^ and USP10-B^KO^ mice were immunized with the SARS-CoV-2 RBD nanoparticle vaccine. The RBD-specific serum IgA and IgG levels, as well as those of the secretory IgA in bronchoalveolar lavage fluid (BALF) were measured via ELISA every two weeks. AID knockout mice served as positive controls. The results showed that the titer of RBD-specific serum IgA and IgG, as well as the secretory IgA in BALF in USP10-B^KO^ mice, was lower than that in USP10-B^WT^ mice (Fig. 4b–d). Meanwhile, the antibody neutralization test for SARS-CoV-2 S protein pseudoviruses indicated that the neutralizing ability of Abs in both serum and BALF-immunized USP10-B^KO^ mice was reduced (Fig.4e, f). All results suggest that USP10 plays a critical role in the immune response to nanoparticle vaccines.

### BCR and TLR1/2 activation synergistically inhibits AID degradation in the nucleus

**Fig. 5:**
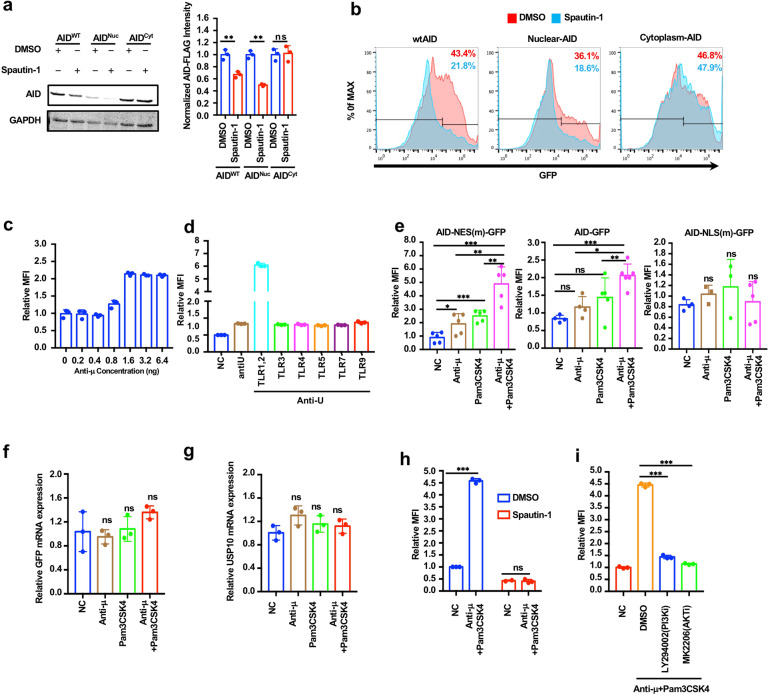
BCR and TLR2 activation synergistically inhibited the AID degradation in the nucleus. **a**, **b** USP10 mainly regulates the protein abundance of AID in the nucleus. **a** Western blot indicated Spautin-1(10 μM) treatment downregulated the nuclear AID (F193A-L196A-AID) in the 293 T cells. **b** FACS indicated Spautin-1(10 μM) treatment downregulated the nuclear AID (F193A-L196A-AID) in the 293 T cells. **c** BCR stimulation slightly upregulated AID-GFP signal in the nucleus although at very high concentrations in the Ramos B Cells. **d** The toll-like receptor stimulator screen indicated TLR1/2 and BCR co-stimulation upregulated the nuclear AID-GFP signal in the Ramos B cells. The concentration of all the toll-like receptor stimulators was 10 μM, the concentration of anti-μ was 1 ng. **e** FACS indicated TLR1/2 with BCR co-stimulation had effects on yhr wild-type AID and nuclear [AID-NES(m)-GFP] but not the cytoplasm AID [AID-NLS(m)-GFP]. **f**, **g** RT-qPCR indicated that TLR1/2 and BCR co-stimulation had no effect on the GFP mRNA transcripts and USP10 mRNA transcripts. **h** The effect of TLR1/2 and BCR on nuclear AID was USP10-dependent. AID-NES(m)-GFP Ramos cells were pretreated with Spautin-1 (10 μM) for 12 h, after anti-μ and Pam3Csk4 co-stimulation. **i** The effect of TLR1/2 and BCR on the nuclear AID was PI3K-AKT dependent. AID-NES(m)-GFP Ramos cells were pretreated with PI3K inhibitor LY294002 (10 μM) or AKT inhibitor MK2206 (10 μM) for 12 h, after the anti-μ and Pam3Csk4 co-stimulation. DMSO was set as a negative control. Data were representative of multiple experiments. *P < 0.05, **P < 0.01, ***P < 0.001

AID is a nucleus–cytoplasm shuttling protein that can be degraded in the nucleus via ubiquitin-dependent or -independent proteasome pathways.^39–41^ As AID is located in the cytoplasm in the resting state of B cells, we investigated whether USP10 regulates the protein abundance of AID in the cytoplasm or nucleus. We constructed an F193A-L196A-AID-expressing plasmid, which disrupted the AID nuclear export signal (NES), and a V18S-AID-expressing plasmid, which disrupted the AID nuclear localization signal (NLS). These two constructs and wild-type AID were separately transfected into 293 T cells following treatment with the USP10 inhibitor spautin-1. Western blot and FACS analyses showed that both the wild-type AID and nuclear AID levels (F193A-L196A-AID) decreased when treated with spautin-1, while the cytoplasmic AID (V18S-AID) level was not affected (Fig. 5a, b). These results indicated that USP10 mainly inhibited AID degradation in the nucleus and affected the AID abundance in the cytoplasm.

The concentration of nuclear AIDs is important. Although various mechanisms have been proposed for the regulation of AID expression and activity, it is not known whether any external signal directly affects AID activity in the nucleus where AID performs its major function. Based on the above findings, we hypothesized that there could be an external signal regulating the stability of AID in the nucleus through USP10. To this end, we constructed a stable AID-NES(m)-GFP-expressing Ramos B cell line that expresses AID-GFP in the nucleus. As BCR is an important signal of B cells and we previously found that BCR could slightly upregulate nuclear AID abundance (Supplemental Fig. 4b), we first tested the effect of BCR on nuclear AID protein expression by adding an anti-μ antibody, which slightly upregulated AID-GFP expression in the nucleus at high concentrations (Fig. 5c). Alternatively, as Toll-like receptors (TLRs) often act in conjunction with BCR and play a key role in B cell development and the immune response,^52^ we hypothesized that there could be a certain kind of TLR that affects AID degradation in combination with BCR. To this end, a variety of TLR stimulators in combination with anti-μ antibodies were added to AID-NES(m)-GFP Ramos B cells. We found that the TLR1/2 activator Pam_3_Csk_4_ treatment with anti-μ antibody significantly upregulated the nuclear AID-GFP abundance in Ramos B cells (Fig. 5e). To further explore whether the synergistic effects of anti-μ antibody and Pam_3_Csk_4_ on AID in the cytoplasm, we generated the AID-NLS(m)-GFP- or AID-GFP-expressing Ramos B cells. The results showed that anti-μ antibody and Pam_3_Csk_4_ co-stimulation affected the abundance of wild-type AID and AID-NES(m)-GFP, but not AID-NLS(m)-GFP. Meanwhile, the anti-μ antibody and Pam_3_Csk_4_ co-stimulation did not affect GFP mRNA transcripts and USP10 mRNA transcripts, indicating that the effect of pam_3_CSK_4_ and anti-μ on nuclear AID abundance occurred in a post-translational manner (Fig. 5f, g).

To further confirm the role of USP10 in anti-μ antibody and Pam_3_Csk_4_ co-stimulation, we pretreated AID-NES(m)-GFP-expressing Ramos cells with the USP10 inhibitor spautin-1 for 12 h, followed by the anti-μ antibody and Pam3Csk4 co-stimulation. The results showed an inhibitory effect on AID-NES(m)-GFP abundance (Fig. 5h). These results indicated that the effect of anti-μ antibody and Pam3Csk4 on nuclear AID was likely USP10-dependent.

Furthermore, as the BCR signal usually activates the downstream PI3K-AKT pathway, the role of the PI3K-AKT pathway in anti-μ antibody and Pam_3_Csk_4_ stimulation was examined by using the PI3K inhibitor LY294002 or AKT inhibitor MK2206.^53,54^ AID-NES(m)-GFP Ramos cells were pretreated with these inhibitors, followed by the anti-μ antibody and Pam_3_Csk_4_ co-stimulation. The results showed that anti-μ antibody and Pam_3_Csk_4_ could no longer upregulate nuclear AID in inhibitor-pretreated cells, indicating that the effect of these cytokines on nuclear AID was PI3K-AKT pathway-dependent (Fig. 5i).

### BCR and TLR1/2 activation synergistically inhibited AID degradation in the nucleus through Akt-mediated USP10 T674 phosphorylation and the nuclear translocation

**Fig. 6:**
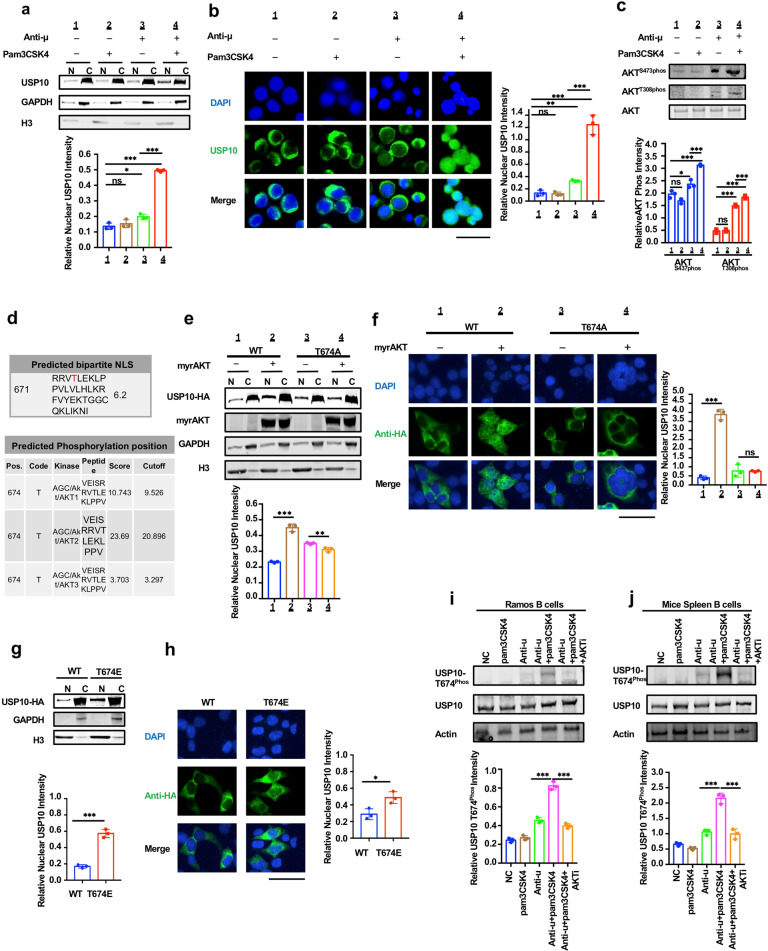
BCR and TLR2 activation synergistically inhibited AID degradation in the nucleus through Akt-mediated USP10 T674 phosphorylation and nuclear translocation. **a**, **b** TLR1/2 and BCR co-stimulation promoted USP10 nuclear translocation. **a** Nucleus–cytoplasm separation experiment. **b** The immunofluorescence assay. **c** Western blot showed that TLR1/2 and BCR co-stimulation enhanced AKT S437 phosphorylation and T308 phosphorylation compared to anti-μ stimulation alone in Ramos B cells. **d** The 671-705 severed as a nuclear localization signal (NLS) of USP10. The T674 in USP10 NLS may be an AKT phosphorylation site. **e**, **f** The myrAKT increased USP10 nuclear translocation but not T674A USP10. **e** Nucleus–cytoplasm separation experiment, H3 as was used as a nuclear loading control. **f** Immunofluorescence assay. **g**, **h** T674E USP10 performed more nuclear distribution compared with wild-type USP10. **g** Nucleus–cytoplasm separation experiment, H3 as was used as a nuclear loading control. **h** Immunofluorescence assay. **I**, **j** Western blot indicated TLR1/2 and BCR co-stimulation promoted USP10 T674 phosphorylation, but not when AKT inhibitor treatment. **i** in Ramos B cells, **j** in Mice spleen B cells. Data were representative of multiple experiments. *P < 0.05, **P < 0.01, ***P < 0.001. The scale bar in image data is 50 μm

USP10 is a cytoplasmic protein characterized by a nucleus–cytoplasm shuttle. Phosphorylation plays an important role in regulating this shuttle activity.^42^ As the above results indicate that USP10 regulates the protein level of AID by inhibiting its degradation in the nucleus, we speculated that whether anti-μ antibody and Pam_3_Csk_4_ co-stimulation increased the nuclear translocation of USP10. To confirm this hypothesis, nuclear–cytoplasmic separation and immunofluorescence assays were performed. The results showed that anti-μ antibody treatment slightly increased USP10 nuclear translocation, but co-treatment with anti-μ antibody and Pam_3_Csk_4_ significantly increased USP10 nuclear translocation in Ramos B cells (Fig. 6a, b).

TLRs, except for TLR3, function mainly through the myeloid differentiation factor (Myd88) as their adapter.^55^ Myd88 was reported to enhance AKT signaling in cells.^56^ Thus, we wondered whether Pam_3_Csk_4_ treatment enhanced BCR-mediated activation of AKT signaling. To confirm this hypothesis, AKT S437 phosphorylation and T308 phosphorylation signals were detected by western blotting. The results showed that the additional pam_3_CSK_4_ treatment enhanced AKT S437 phosphorylation and T308 phosphorylation compared with the anti-μ antibody stimulation alone (Fig. 6c).

To explore whether and how the phosphorylated AKT further affects the downstream USP10 nuclear translocation, we first predicted the AKT phosphorylation position in USP10 using cNLS Mapper tools (http://nls-mapper.iab.keio.ac.jp/cgi-bin/NLS_Mapper_form.cgi) and Group-based Prediction System 5.0.^57,58^ The results showed that the amino acids at 671-705 served as a nuclear localization signal (NLS) of USP10, and the T674 within the USP10 NLS might be an AKT phosphorylation site (Fig. 6d). Thus, we assumed that T674 phosphorylation might regulate USP10 nuclear translocation. To test this hypothesis, we constructed a T674E USP10 mutated plasmid, which mimics the phosphorylation state of this site. The nuclear-cytoplasm separation assay and immunofluorescence assay showed that T674E USP10 had more nuclear distribution compared to the wild-type USP10 (Fig. 6g, h). To further confirm whether T674 phosphorylation and nuclear translocation of USP10 are regulated by AKT, we constructed a T674A USP10 mutant that prevented the phosphorylation at T674. A plasmid expressing MyrAKT, which is a type of continuously activated Akt, was co-transfected with plasmids expressing USP10 or T674A USP10 in 293 T cells. Western blot and immunofluorescence assays showed that the MyrAKT increased USP10 nuclear translocation, but not T674A USP10 (Fig. 6e, f). In addition, the in vitro kinase assay confirmed that AKT directly phosphorylates wild-type USP10 peptide, but not T674A USP10 peptide (Supplementary Fig. 4c). Moreover, a special detection with the phospho-(Ser/Thr) Akt substrate antibody confirmed that USP10 phosphorylation was directly related to AKT (Supplementary Fig. 4f). To further clarify that anti-μ antibody and Pam_3_Csk_4_ co-treatment promoted T674 phosphorylation of USP10, we first generated an antibody that specifically recognizes the phosphorylation site of USP10 by immunizing rabbits with the phosphorylated and sequence-homologous polypeptides (Supplementary Fig. 4a). Western blot analysis of Ramos B cells and mouse spleen B cells showed that the co-stimulation of anti-μ antibody and Pam_3_Csk_4_ promoted USP10 T674 phosphorylation, but not when AKT inhibitor was used (Fig. 6i, j). A previous study indicated that nuclear translocation of USP10 was regulated by ATM-mediated phosphorylation of USP10 at Thr42 and Ser337.^42^ We compared the effects of T674E USP10 and T42E-S337D-USP10 on nuclear AID degradation using a dendra2 decay assay. The results indicated that T674E USP10 was more likely to regulate AID concentration in the nucleus than T42E-S337D-USP10 (Supplementary Fig. 4d). Moreover, both Thr42 and Ser337 sites in USP10 were found to not be of the Akt phosphorylation motif via analysis with the Group-based Prediction system 5.0 (Supplementary Fig. 4e). Collectively, these results indicated that BCR and TLR2 synergistically enhance Akt activity and promote USP10 T674 phosphorylation located within the NLS domain and, therefore, the nuclear import of USP10, consequently inhibiting the AID degradation in the nucleus.

### Pam_3_Csk_4_ helped to enhance the antibody response to the SARS-CoV-2 RBD nanoparticle vaccine

**Fig. 7:**
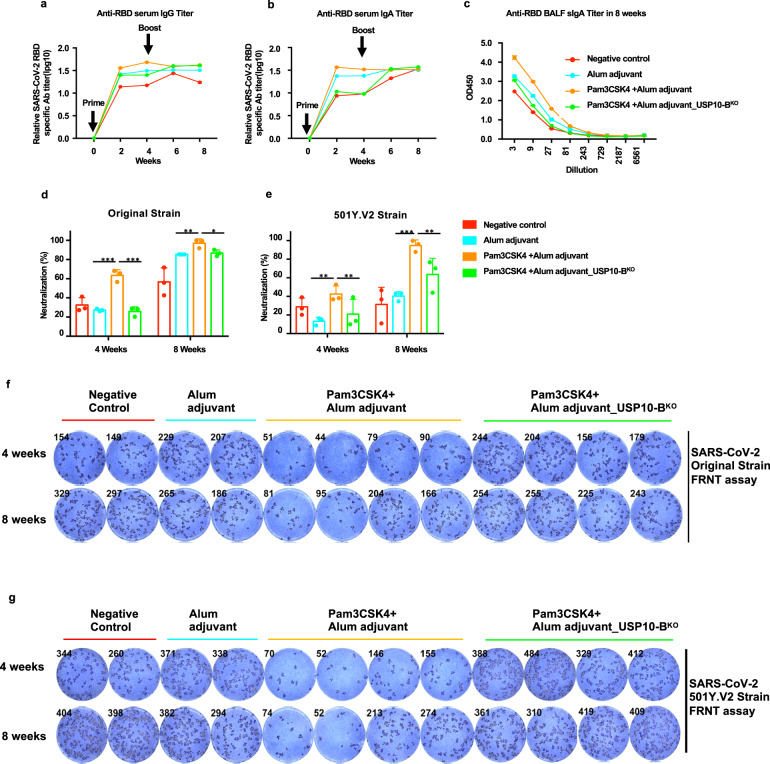
Pam3CSK4 enhanced the antibody response to the SARS-CoV-2 RBD nanoparticle vaccine. The USP10-B^WT^, USP10-B^KO^ mice were prime/boost-vaccinated with 5 ug of the SARS-CoV-2 RBD nanoparticle vaccines at week 0 and week 4. The Alum or Alum+Pam_3_CSK_4_ is used as adjuvants. Serum was collected every 2 weeks. All mice were euthanized at week 10. **a**, **b** The RBD-specific IgG, IgA relative titers at each week were plotted as a time-course curve. **c** The bronchoalveolar lavage fluid (BALF) was collected from euthanized mice in week 10. RBD-specific sIgA titers plotted as OD value versus dilution factors. **d**, **e** The neutralization of sera in immunized mice was detected with pseudotyped-SARS-CoV-2 original strains and 501Y.V2 strains. The neutralizations at dilution of 1:200 of serum were shown. **f**, **g** The serum neutralization to authentic SARS-CoV-2 of each vaccine group was determined by FRNT assay with anti-SARS-CoV-2 N protein antibody staining. The SARS-CoV-2 original strain and 501Y.V2 strain were separately used. The representatives of FRNT spot wells within 1:20, 1:200, or 1:2000 dilution groups were shown

Pam_3_Csk_4_ has been used as a vaccine adjuvant.^59^ To explore the effect of Pam_3_Csk_4_ on the SARS-CoV-2 RBD particle vaccine immune response, USP10-B^WT^ and USP10-B^KO^ mice were immunized with adjuvants in combination with the SARS-CoV-2 RBD particle vaccine. The RBD-specific IgG and IgA antibodies were measured by ELISA every two weeks. The results showed that Pam_3_Csk_4_ facilitated the production of RBD-specific IgA and IgG antibodies (Fig. 7a, b). In addition, Pam_3_Csk_4_ facilitated RBD-specific sIgA antibody production in BALF, while USP10 knockout in B cells impaired this effect (Fig. 7c). Moreover, the pseudotype-virus neutralization test and authentic SARS-CoV-2 FRNT assay showed that Pam_3_Csk_4_ enhanced the neutralization ability of sera against the viruses. (Fig. 7d, f). Interestingly, Pam_3_Csk_4_ also significantly increased the neutralization ability of sera against the SARS-CoV-2 501Y.V2 strain with the E484K mutation after immunization (Fig. 7e, g). Therefore, Pam_3_Csk_4_ could be used as an excellent adjuvant to enhance the immune effect of the SARS-CoV-2 RBD particle vaccine.

## Discussion

A sufficient concentration of AID in the nucleus is required to efficiently facilitate CSR and SHM. However, nuclear AIDs are tightly regulated. Most AID is retained in the cytoplasm by interacting with eEF1A and HSP90 to hinder the passive diffusion of AID into the nucleus.^33,34,36,60,61^ Once AID is imported by GANP and/or importin, it is quickly exported back to the cytoplasm by CRM1 or degraded by the proteasome pathway in the nucleus.^28,37,40,62,63^ Moreover, AID enters the nucleus in a short pulse manner to synchronize both with the factors that respond to AID-initiated damage and those that regulate the transcription of genes affecting AID expression.^64^ In addition, the activity and concentration of nuclear AID are regulated by the cell cycle.^65^ However, the direct regulation of nuclear AID abundance by extracellular signal(s) has not been elucidated. In this study, we demonstrated that USP10 is a specific deubiquitinase for AID by removing the ubiquitins of AID and inhibiting its degradation in the nucleus. We demonstrated how USP10, as a shuttle protein, stabilizes AID in the nucleus. We also clarified that T674 phosphorylation within the NLS domain of USP10 regulates the import of USP10 from the cytoplasm to the nucleus. Interestingly, the BCR-PI3K-Akt signaling pathway activates AKT and phosphorylates the T674 site of USP10 and the TLR1/2 signal, which significantly enhances signal transduction. As a result, T674^phos^-USP10 was imported into the nucleus and inhibited the degradation of nuclear AID to ensure efficient CSR and SHM (Supplementary Fig. 6). Therefore, this study has demonstrated a direct connection between the extracellular signals and nuclear AID abundance, and deubiquitinase plays a key role in this important signal transduction pathway.

A previous study revealed that the concentration of AID is tightly regulated at multiple layers, including transcription, post-transcription, and post-translation. AID accumulates in the cytoplasm during storage, while the AID in the nucleus performs its major function. The enhancement of AID expression via regulation of transcription or post-transcription may only increase the amount of AID in storage, but not the amount at the worksite. PTM directly alters the fate and activity of the protein required for its function in situ. It has been shown that BCR signaling alone exerts a paradoxical phenomenon by upregulating or downregulating *Aicda* transcription.^16,66,67^ It is generally believed that BCR needs other stimuli, such as IL-4, CD40, TLR4, and TLR9, to synergistically enhance *Aicda* transcription.^16,67,68^ To the best of our knowledge, the BCR/TLR1,2- USP10-AID pathway is the only pathway that directly regulates the nuclear AID abundance rather than the total *AICDA* expression. In particular, the phosphorylation of USP10 in its NLS domain by BCR-TLR1/2-PI3K-Akt integrates the dual signals from the interaction between TLR and its ligand, or BCR and antigen on the cell surface, facilitating its translocation into the nucleus and transmitting signals into the nucleus to regulate the abundance of AID. Once the dual stimulations weaken, the nuclear abundance of AID will decrease correspondingly. Compared with other identified signal transduction pathways from BCR or TLR to AID expression, this pathway is more precise and economical to directly alter the concentration of AID on its worksite which is in the nucleus.

The TLR family plays a critical role in initiating innate inflammatory responses and promoting adaptive immune responses. Most TLRs transmit their signals via MyD88.^69^ MyD88 elicits transcriptional alterations mainly through the activation of NF-κB or other transcription factors. The efficient stimulation of TLRs in B cells is dependent on the type of antigen.^52^ When the mice were immunized with soluble proteins with TLR ligands, the magnitude of the IgG responses was comparable between B cell MyD88-deficient mice and wild-type controls. However, when the mice were immunized with virus-like particles (VLPs) containing CpG-oligodeoxynucleotides (a TLR9 ligand), the deficiency of MyD88 in B cells caused a 30-fold decrease in IgG response.^70^ The present study further provides a mechanism for the synergistic action of particle antigen and TLR stimulation during the immune response of B cells. However, why TLR1/2, rather than other TLRs, plays a leading role in this process remains to be elucidated. The synthetic bacterial lipopeptide Pam3-Cys-ser-Lys4 (Pam3CSK4) is a synthetic tripalmitoylated bacterial lipopeptide that has been widely used as a potent adjuvant for various vaccines, including a sublingual allergy vaccine, flu vaccine, and leishmaniasis vaccine.^71^ A previous study showed that Pam3CSK4 combined with TLR-independent adjuvant MF59 increased the immunogenicity of a trivalent influenza seasonal subunit vaccine in mice, supporting the role of pam3CSK4 as an effective adjuvant in the subunit vaccine.^59^ This study indicated that Pam3CSK4 could be a promising adjuvant for the SARS-CoV-2 RBD nanoparticle vaccine.

SARS-CoV-2 neutralizing monoclonal antibodies obtained from individuals during the early convalescence period showed a low level of somatic mutations and defects in germinal center formation.^72,73^ The neutralizing antibodies develop in most individuals after infection but decay between 1.3 and 6.2 months. However, a recent study indicated that the anti-SARS-CoV-2 memory B cell response evolves during the first 6 months after infection with an accumulation of Ig somatic mutations and the production of antibodies with increased neutralizing breadth and potency because of the persistence of antigens.^72^ The concentration upregulation of nuclear AID may promote *IgH* SHM and increase the neutralizing breadth of SARS-CoV-2 mutated strains. The design of the vaccine aimed to improve the nuclear abundance of AID and increase SHM via adjuvant, which may broaden the neutralization spectrum to prevent the infection of mutant SARS-CoV-2, such as E484K mutated virus strains that resist neutralizing antibodies after vaccine immunization.^74,75^

HIV-1 bnAbs have several characteristics: (1) they are produced in a specific antibody gene; (2) the CDR L3 and H3 regions of the antibody have a specific length and conserved motif; and (3) compared with the common antibodies, the mutation frequency of the germline is very high.^76,77^ These characteristics mean that HIV-1 bnAbs are only produced in a small number of elite patients and are quite difficult to induce with vaccine immunity.^78,79^ The essential condition for the development of bnAbs is a high frequency of mutations in the antibody germline gene, which requires high intensity and prolonged germinal center response.^48,80,81^ Therefore, improving the mutation frequency of antibody genes is the key to successfully inducing bnAbs in a short period of time. The results of this study confirmed that the frequency of SHM in *IgH* was improved when the AID abundance in the nucleus was increased by manipulating USP10. In addition, using VRC01-knock-in mice, we confirmed that the presence of USP10 significantly affected the development of VRC01-like antibodies. Therefore, the vaccine adjuvant that regulates USP10 nuclear translocation could increase the AID abundance in the nucleus and thus improve the probability of bnAbs development after immunization with the HIV-1 vaccine.

In summary, we identified a novel pathway that rapidly and precisely regulates the concentration of nuclear AID in B cells. In particular, the BCR/TLR1-2 receptor transmits the extracellular signaling to the intracellular PI3K-AKT pathway and activates USP10 nuclear import to maintain the concentration of nuclear AID. Manipulation of this signaling pathway during immunization could significantly enhance the humoral immune response to the vaccine. Therefore, this study provides theoretical guidance for the design of vaccines against SARS-CoV-2 or HIV-1.

## Materials and methods

The information of all experimental materials used in this study are listed in the supplemental materials.

### Animals

Mice were bred and housed under conventional conditions at Sun Yat-sen University Laboratory Animal Center in accordance with the guidelines and principles of the Institutional Animal Care and Use Committee (IACUC) of Sun Yat-sen University. Experiments were carried out using age- and gender-matched mice in strict accordance with good animal practice as defined by the National Center for the Replacement, Refinement & Reduction of Animals in Research (NC3Rs). Mice serum was sampled by extracting the eyeball blood after mice were anesthetized with isoflurane. The mice were euthanized if their weight loss was less than 75%. All efforts were employed to avoid the pain of mice.

AID-KO mice were provided by Tasuku Honjo through Riken BRC (RBRC: 00897). CD19-cre (B6.129P2 (C) Cd19tm1 (cre) cgn/J; Stock No 006785), AID-GFP (C57BL/6-Tg (Aicda/EGFP) 1Rcas/J; Stock No 018421), and VRC01 (B6(Cg) Ightm3.1 (VRC01) Nemz/J; Stock No 029584) mice were purchased from Jackson Laboratory. USP10-CKO mice were constructed by Shanghai Model Organisms through CRISPR-Cas9 technology. The mice were mated with CD19-cre mice to produce USP10-BKO (usp10 specifically knockout in B cells) Mice. The usp10 gene knockout and validation strategies were shown in Supplementary Fig. 5.

### Cells

The HEK 293 T cells were obtained from ATCC and maintained in DMEM supplemented with 10% fetal bovine serum (Gibco) plus 1% penicillin-streptomycin at 37 °C with 5% CO_2_. The CH12 cells and mice primary spleen cells were maintained in 1640 medium supplemented with 10% fetal bovine serum and 1% penicillin-streptomycin and 2-ME (55um, Gibco) at 37 °C with 5% CO_2_. The CH12 cells are a gift from Tasuku Honjo (Kyoto University, Japan) through Hu Wenjun (Kyoto University, Japan) and Meng Feilong (Shanghai Institute of Biochemistry and Cell Biology, CAS). The Ramos Ra.1 cells were obtained from ATCC and maintained in 1640 medium supplemented with 20% fetal bovine serum and 1% penicillin-streptomycin at 37 °C with 5% CO2. All cell lines were confirmed mycoplasma negative.

### Western blotting and co-immunoprecipitation

The cells were lysed in ice-cold RIPA lysis buffer containing a protease inhibitor mixture (Sigma) for 30 min at 4 °C. The lysates were subjected to Western blot. For co-immunoprecipitation, the 293 T cells were cultured in a 60-mm-diameter plate and transfected with various indicated plasmids. Forty-eight hours later, the cells were collected and disrupted using a lysis buffer containing a protease inhibitor mixture (Sigma) and PMSF for 30 min at 4 °C. The cell lysates were separated by centrifugation at 12,000 rpm for 15 min at 4 °C. Anti-HA agarose beads (Sigma) or Anti-FLAG agarose beads (Sigma) were mixed with the cell lysates and incubated at 4 °C for 4 h or overnight. If immunoprecipitated endogenous USP10, the rabbit anti-USP10 (Abcam) or rabbit IgG and Protein G agarose beads were used. The beads were subsequently washed four times with the cold lysis buffer and diluted with a gel loading buffer. The immunoprecipitated samples were analyzed by SDS-PAGE, followed by western blotting. Bands were immunoblotted with indicated antibodies and IRDye secondary antibodies (LI-COR) and visualized with the Odyssey infrared imaging system (LI-COR).

### Transfection

The plasmids or chemically synthesized siRNAs (Ribo Bio, Guangzhou, China) were transfected into the 293 T cells using Lipofectamine 2000 (Invitrogen) according to the manufacture’s protocol. For CH12 cells, the electrotransfection (Neon system, Thermo Fisher) was used according to the manufacture’s protocol.

### Monitor protein degradation with photoactivatable fluorescent technology

The photoactivatable fluorescent assay was performed as described previously.^41^ Briefly, a green-to-red photoconvertible fluorescent protein dendra2 was fused to the C-terminus of AID. The AID-dendra2 expressed 293 T cells were exposed to the 405 nm surface light for 2 min to convert green fluorescence to red fluorescence. The red fluorescence images were collected by a fluorescence microscope each 2 h. These images were analyzed by the Image J software.

### Mass spectrometry analysis

The silver-stained bands of interest were excised into a clean tube. The samples were digested with trypsin using the in-gel digestion method. In detail, as previously described.^82^ All samples were analyzed on a Thermo Scientific Q EXACTIVE mass spectrometer coupled with an EASY n-LC 1000 liquid chromatography (Thermo Fisher) system and a nanoelectrospray source.

### Ubiquitination assay

The transfected cells were lysed with RIPA buffer containing N-Ethylmaleimide (NEM), PMSF, protease inhibitor mixture after MG132(10um) treatment 8 h. The cell lysates were separated by centrifugation at 12,000 rpm for 15 min at 4 °C. Anti-FLAG agarose beads (Sigma) were mixed with the cell lysates and incubated at 4 °C for 4 h. The beads were then washed four times with cold 300 mM NaCl STN buffer and eluted with a gel loading buffer. The samples were analyzed by SDS-PAGE, followed by western blotting.

### Class switching assay

CD43^−^ naïve splenic B lymphocytes were purified by magnetic separation (MACS, Miltenyi Biotec). The cells were maintained at 1–3 × 10^6^ cells/ml in standard culture medium and were treated with 5 ng/ml IL-4 and 25 ug/ml lipopolysaccharide (LPS) so that CSR would be induced to IgG1; with 25 ug/ml LPS, 5 ng/ml TGF-beta, and 10 ng/ml anti-IgD for induction of CSR to IgA. The CH12 cells were treated with 5 ng/ml IL-4, 0.2 ug/ml anti-CD40, and 5 ng/ml TGF-beta so that CSR would be induced to IgA.

### Mutation analysis

The mutation analysis experiment was performed as described previously.^83^ In brief, an intronic sequence 3′ to the JH4 exon of IgH and the Vh186.2 sequences were PCR-amplified from genomic DNA extracted from GC B cells. The PCR products were inserted into the pMD20-T vector and sent to the sanger sequence.

### Affinity maturation analysis

To quantify NP-specific antibodies, the serum of NP-CGG immunized mice were sampled on days 7, 14, and 21. NP-specific high-affinity and low-affinity antibodies were captured on plates coated with 10 ug/ml NP_2_-BSA or NP_23_-BSA, respectively.

### Flow cytometry

Cell surface marks were stained by using fluorophore-conjugated antibodies. The flow cytometry antibodies used in analysis and sorting can be found in the Supplemental Table. Flow cytometry and cell sorting were conducted on the LSR II and Aria II cytometers (BD bioscience), respectively. The data were analyzed with FlowJo software.

### Immunofluorescence

The cells on coverslips or the tissues were fixed with paraformaldehyde (4%). For intracellular staining, the permeabilization solution was added and incubate for 3–5 min. Add blocking buffer (5% BSA) and incubate 60 min at room temperature for blocking. The diluted primary antibody and fluorochrome-conjugated secondary antibody were used to stain the target protein. The Immunofluorescence images were captured by a fluorescence microscope.

### RT-PCR

RNA was extracted with TRIZOL according to the manufacturer’s instructions (Invitrogen). Reverse transcription reactions were conducted with the PrimeScript RT reagent Kit (TaKaRa). Quantitative PCR was conducted with the SYBR Premic ExTaq Kit (TaKaRa) on a CFX96 Real-Time System (Bio-Rad). Mouse GAPDH mRNA was measured as endogenous controls.

### In vitro kinase assay

The luciferase-based ADP-Glo kinase assay (Promega[V6930]) was used to measure kinase activity by quantifying the amount of ADP produced during a kinase reaction (see ADP-Glo kinase assay technical protocol). In detail, wild-type USP10 peptide or T674A USP10 peptide (5 μM) were diluted in 1 X Kinase Reaction Buffer A (containing 40 mM Tris, pH 7.5, 0.1 mg/mL BSA, and 20 mM MgCl2) . The peptide was serially diluted and added to each well in duplicate. Then, 2.5 μl of 100 μM ATP (Promega) and 0.25 μg AKT (Sino Biological) was added to a 25 μL reaction mixture. The kinase cocktail reaction occurred for 20 min at room temperature (25 μl/well). An equal volume of ADP-Glo reagent was added at room temperature and allowed to equilibrate for 40 min to terminate the kinase reaction and deplete the remaining ATP. Finally, the kinase detection reagent (50 μl) was added at room temperature and allowed to equilibrate for 50 min to convert ADP to ATP. A Promega GLOMAX luminometer reader was used for the detection of luminescence.

### CRISPR-Cas9 mediated silencing of USP10 expression in CH12 cells

The Cas9 was minutely modified to improve fidelity as described previously.^84^ To generate the USP10 knockout CH12 cell lines, an USP10-specific sgRNA was cloned into the pX458 plasmid. The constructs were electrotransfected to CH12 cells by using the Neon electrotransfection system (Thermo Fisher) according to the manufacturer’s protocol. After 24 h, the single GFP positive CH12 cell was sorted by FACS and maintained in conditional medium (50% used medium + 50% new medium) for cell amplification. The effect of USP10 silence in CH12 was confirmed by DNA electrophoresis and western blot (Supplemental Fig. 4).

### Authentic SARS-CoV-2 assay, FRNT50 assay, pseudotyped virus neutralization assay

The method was consistent with our previously published works.^51,85^ Briefly, an original SARS-CoV-2 strain named nCoV-19/CHN/SYSU-IHV/2020 strain (accession ID on GISAID: EPI_ISL_444969) was isolated by our lab and a South African SARS-CoV-2 strain (501Y.2) (GDPCC-nCOV-84), which was isolated from a South Africa traveler by the Guangdong Center for Disease Control, were used. For FRNT (Focus Reduction Neutralizing test) assay, Vero E6 cells were seeded in 96-well plates at a density of 2 × 10^4^ cells per well. The cells were incubated with a virus/serum mixture for 1 h at 37 °C. Then, the plates were then incubated for 1 h at 37 °C. The supernatant was removed and cells were overlaid with DMEM medium containing 1.6% CMC. The 96-well plates were placed in the incubator and incubated for 24 h. On the second day, the supernatant was removed completely. Cells in each well were fixed with 200 μL of 4% paraformaldehyde for 12 h at 4 °C and subsequently incubated with 100 μL of PBS containing 0.2% Triton X-100 and 1% BSA for 30 min. Cells were then incubated with 50 μl of diluted primary antibody against SARS-CoV-2 nucleocapsid (N). For pseudotyped virus neutralization assay, HEK293T cells were co-transfected with a packaging plasmid psPAX2, a luciferase-expressing plasmid pHIV-Luciferase, and a plasmid expressing spike proteins of SARS-CoV-2. Serially diluted serum of mice was mixed with the pseudotyped viruses and incubated at 37 °C, 5% CO_2_ for 1 h. The serum/virus mixtures were added into wells containing 1 × 10^4^ hACE2-HeLa cells and went on culturing for 48 h. The serum neutralization efficiency was measured via detecting luciferase activity in cells.

### SARS-CoV-2 RBD nanoparticle and eOD-GT8-60mer nanoparticle construction

The method was consistent with our published work.^51^ Briefly, for SARS-CoV-2 RBD nanoparticle. SC-Ferritin was expressed and purified from Escherichia coli (E.coli). ST-RBD was expressed and purified from CHO-S cells. SC-Ferritin and ST-RBD were mixed and incubated in Tris buffer to facilitate ST/SC irreversible conjugation. The conjugated nanoparticles were separated with SEC and concentrated by ultrafiltration device. For eOD-GT8-60mer nanoparticle. LS-eOD-GT8-60mer was expressed and purified from 293 F cells. Briefly, the DNA sequence of 6 X His-labeled SP-LS-eOD-GT8-60mer was cloned into a pcDNA3.1 vector. The constructed plasmid was transfected into 293 F cells. After seven days, the supernatant was collected and centrifuged to discard cell debris. The cleared supernatant was passed through Ni-NTA agarose to enrich the His-labeled target protein and then eluted with Tris buffer containing imidazole. The purified protein was concentrated and the buffer was replaced with a regular Tris buffer. The protein concentration was determined by the BCA assay method. The Coomassie Blue staining was performed to confirm its purity.^49^

### Statistical analysis

Unless otherwise indicated, values are reported as the mean ± SEM, Values of *p* < 0.05 were considered significant. Statistical significance between two samples was determined by using the two-tailed student’s *t*-test. *P* values are denoted in figures by *p < 0.05; **p < 0.01; and ***p < 0.001

## Supplementary information

Sigtrans_Supplementary_Materials

## Data Availability

All data that support the findings of this study are available within the paper, supplementary information, or available from the corresponding author upon reasonable request. LC-MS AID associated proteins data and BCR data had uploaded on Figshare (10.6084/m9.figshare.17058332.v1, 10.6084/m9.figshare.17058602.v1).
