# Supplementary material for: USP10 regulates B cell response to SARS-CoV-2 or HIV-1 nanoparticle vaccines through deubiquitinating AID
Source: Signal Transduct Target Ther. 2022 Jan 4;7:7. doi: 10.1038/s41392-021-00858-z (PMC8724756; doi:10.1038/s41392-021-00858-z)
Supplement: Supplementary file 1 — Sigtrans_Supplementary_Materials [file 41392_2021_858_MOESM1_ESM.docx]

Supplementary Materials for

USP10 Regulates B cell Response to SARS-CoV-2 or HIV-1 Nanoparticle Vaccines through Deubiquitinating AID

Yuewen Luo1#, Xiantao Zhang#, Ran Chen#, Rong Li, Yang Liu, Junsong Zhang, Qin liu, Meijun Si, Jun Liu, Bolin Wu, Xuemei Wang, Shijian Wu, Yiwen Zhang, Xu Zhang, Deyin Guo, Xin He, Ting Pan, and Hui Zhang*

Correspondence to: zhangh92@mail.sysu.edu.cn

**This PDF file includes:**

Materials and Methods

Supplementary Figure S1-S6

Materials and Methods

Experimental Materials table

| **REAGENT or RESOURCE** | | **SOURCE** | | | | **IDENTIFIER** |
| --- | --- | --- | --- | --- | --- | --- |
| **Antibodies** | | | | | | |
| Mouse monoclonal anti-HA | | MBL | | | | M180-11 |
| Rabbit polyclonal anti-FLAG | | MBL | | | | PM020 |
| Rabbit polyclonal anti-GAPDH | | Proteintech | | | | 10494-1-AP |
| Rabbit polyclonal anti-USP10 | | Abcam | | | | ab70895 |
| Mouse monoclonal anti-AID (Clone L7E7) | | Cell Signaling Technology | | | | #4975S |
| Phospho-(Ser/Thr) Akt Substrate Antibody | | Cell Signaling Technology | | | | #9611 |
| Mouse monoclonal anti-β Actin (Clone 2D4H5) | | Proteintech | | | | 6009-1-lg |
| Mouse monoclonal anti-Flag tag(Clone 1E7B4) | | Proteintech | | | | 66008-2-lg |
| PE-Cy7 Rat monoclonal anti-Mouse IgM(Clone R6-60.2) | | BD Biosciences | | | | #552867 |
| PE Rat monoclonal anti-Mouse IgG1(Clone A85-1) | | BD Biosciences | | | | #550083 |
| FITC Rat monoclonal anti-Mouse IgA(Clone C10-3) | | BD Biosciences | | | | #559354 |
| APC Rat monoclonal anti-Mouse CD45R/B220(Clone RA3-6B2) | | BD Biosciences | | | | #561880 |
| PE Rat monoclonal anti-Mouse CD43(Clone S7) | | BD Biosciences | | | | #561857 |
| GL7 Monoclonal Antibody (GL-7 (GL7)),Alexa Fluor 488 | | eBioscience | | | | 53-5902-82 |
| Anti-Mouse/Rat CD40 (CloneHM40-3) Functional Grade Purified | | eBioscience | | | | 16-1541 |
| Mouse IgD monoclonal antibody (Clone 10.4.22), Functional Grade | | eBioscience | | | | 16-5924-82 |
| Rabbit anti-USP10-T674^phos^ antibody | | this paper | | | | N/A |
| Phospho-Akt (Ser473) (D9E) XP® Rabbit mAb | | CST | | | | #4060 |
| [Phospho-Akt (Thr308) (D25E6) XP®Rabbit mAb](https://www.cellsignal.cn/products/primary-antibodies/phospho-akt-thr308-d25e6-xp-rabbit-mab/13038?site-search-type=Products&N=4294956287&Ntt=akt&fromPage=plp) | | CST | | | | #13038 |
| Akt (pan) (11E7) Rabbit mAb | | CST | | | | #4685 |
| Goat anti-Mouse IgG(H+L) Secondary Antibody, HRP | | Invitrogen | | | | Cat#31430 |
| **Bacterial and Virus Strains** | | | | | | |
| ﻿A SARS-CoV-2 strain named nCoV-19/CHN/SYSU-IHV/2020  strain (accession ID on GISAID: EPI_ISL_444969) | | Our lab | | | | N/A |
| GDPCC-nCOV-84 (SARS-CoV-2_ B.1.351) | | Guangdong Center for Diseases Control | | | | N/A |
| DH5α competent Escherichia coli cells | | N/A | | | | N/A |
| HB101 competent Escherichia coli cells | | N/A | | | | N/A |
| **Chemicals, Peptides, and Recombinant Proteins** | | | | | | |
| Spautin-1 | | Selleck | | S7888 | | |
| MG132 | | Selleck | | S2619 | | |
| Lipopolysaccharides(LPS) | | Sigma-Aldrich | | L2630 | | |
| Rat/Mosue TGF-beta 1/ TGFB1 Protein | | Sino Biological Inc. | | 80116-RNAH | | |
| Recombinant Murine IL-4 | | PeroTech | | #081449 | | |
| Human IFN-γ | | Sino Biological Inc. | | 11725-HNAS | | |
| Human BAFF Protein | | Sino Biological Inc. | | 10056-HNCH-5 | | |
| Human APRIL Protein | | Biolegend | | #591309 | | |
| Human IL-21 | | Sino Biological Inc. | | 10584-HNAE | | |
| Human TGF-β1 | | Sino Biological Inc. | | 10804-H08H1 | | |
| Human IL-10 | | Sino Biological Inc. | | 10947-HNAE-5 | | |
| Human IL-6 | | Sino Biological Inc. | | 10395-HNAE-5 | | |
| Human IL-4 | | Sino Biological Inc. | | 11846-HNAE-5 | | |
| Recombinant Human AKT | | Sino Biological Inc. | | 10763-H08B | | |
| CD43(Ly-48) MicroBeads, mouse | | MACS,Miltenti Biotec | | 130-049-801 | | |
| F(ab')2-Goat anti-Human IgM (H+L) Secondary Antibody, Functional Grade | | eBioscience | | 16-5099-025 | | |
| Pam3CSK4 | | InvivoGen | | tlrl-pms | | |
| LY294002 | | Selleck | | S1105 | | |
| MK2206 | | Selleck | | S1078 | | |
| **Critical Commercial Assays** | | | | | | |
| ProteoSilver^TM^ Plus Silver Stain Kit | | Sigma | | | | PROTSIL2 |
| ADP-Glo kinase assay | | Promega | | | | V6930 |
| EZview™ Red ANTI-FLAG® M2 Affinity Gel | | Sigma | | | | F2426 |
| Protein G Resin | | Genscript | | | | L00209 |
| NuPAGE™ 4-12% Bis-Tris Protein Gels | | Invitrogen | | | | NP0336BOX |
| NuPAGE™ MES SDS Running Buffer | | Invitrogen | | | | NP0002 |
| NE-PER™ Nuclear and Cytoplasmic Extraction Reagents | | Thermo Fisher | | | | 78835 |
| **Experimental Models: Cell Lines** | | | | | | |
| HEK 293T Cells | | N/A | | | | N/A |
| CH12 Cells | | A gift from Tasuku Honjo | | | | N/A |
| Ramos Cells | | ATCC | | | | CRL-1596 |
| **Experimental Models: Organisms/Strains** | | | | | | |
| Mouse: B6.129P2(C)Cd19tm1(cre)cgn/J | Jackson Laboratory | | | | JAX 006785 | |
| Mouse: C57BL/6-Tg(Aicda/EGFP)1Rcas/J | Jackson Laboratory | | | | JAX 018421 | |
| Mouse: B6(Cg)-Ightm3.1(VRC01)Nemz/J | Jackson Laboratory | | | | JAX 029584 | |
| Mouse: USP10 conditional knockout mice | Shanghai Model Organisms | | | | N/A | |
| Mouse:B6.Cg-Aicda<tm1Hon>(N10)/HonRbrc | A gift from Tasuku Honjo through Riken BRC | | | | RBRC:00897 | |
| **Oligonucleotides** | | | | | | |
| siUSP10-1: CAAACAAGAGGTTGAGATA | this paper | | | | | N/A |
| siUSP10-2:  ﻿GAGGAAATGTTGAACCTAA | this paper | | | | | N/A |
| sgUSP10-1：GAGTGAGGGACTACAAGCTAAGG | this paper | | | | | N/A |
| sgUSP10-2: TGTCACGTACATATTTGTCGAGG | this paper | | | | | N/A |
| sgUSP10-3：GTGCACAGTACCCAAACCATTGG | this paper | | | | | N/A |
| sgUSP10-4:  ACCCAGTGGTGGTAGCCGACCGG | this paper | | | | | N/A |
| **Recombinant DNA** | | | | | | |
| pcDNA3.1-hAID-FLAG | | this paper | | | N/A | |
| pcDNA3.1-hUSP10-HA | | this paper | | | N/A | |
| pcDNA3.1-hUSP10(CA)-HA | | this paper | | | N/A | |
| pcDNA3.1-hUSP10(T674E)-HA | | this paper | | | N/A | |
| pcDNA3.1-hUSP10(T42E-S337D)-HA | | this paper | | | N/A | |
| pcDNA3.1-hUSP10(T673E-S337D)-HA | | this paper | | | N/A | |
| pcDNA3.1-hUSP10(T674A)-HA | | this paper | | | N/A | |
| pcDNA3.1-Dendra2 | | this paper | | | N/A | |
| pcDNA3.1-hAID(F193A, L196A)-Dendra2 | | this paper | | | N/A | |
| pcDNA3.1-hAID-Dendra2 | | this paper | | | N/A | |
| pcDNA3.1-Ub-HA | | this paper | | | N/A | |
| pX458-mUSP10 | | this paper | | | N/A | |
| **Software and Algorithms** | | | | | | |
| FlowJo for Mac 10.0.7 | Treeview | | https://www.flowjo.com/ | | | |
| GraphPad Prism 7 for Mac | Graphpad | | https://www.graphpad.com/scientific-software/prism/ | | | |
| [ImageJ bundled with Java 1.8.0_172](http://wsr.imagej.net/distros/osx/ij152-osx-java8.zip) for mac | NIH | | https://imagej.nih.gov/ij/ | | | |
| Group-based Prediction System v5.0 | CUCKOO Workgroup | | http://gps.biocuckoo.cn/ | | | |
| cNLS Mapper tools | N/A | | <http://nls-mapper.iab.keio.ac.jp/cgi-bin/NLS_Mapper_form.cgi> | | | |


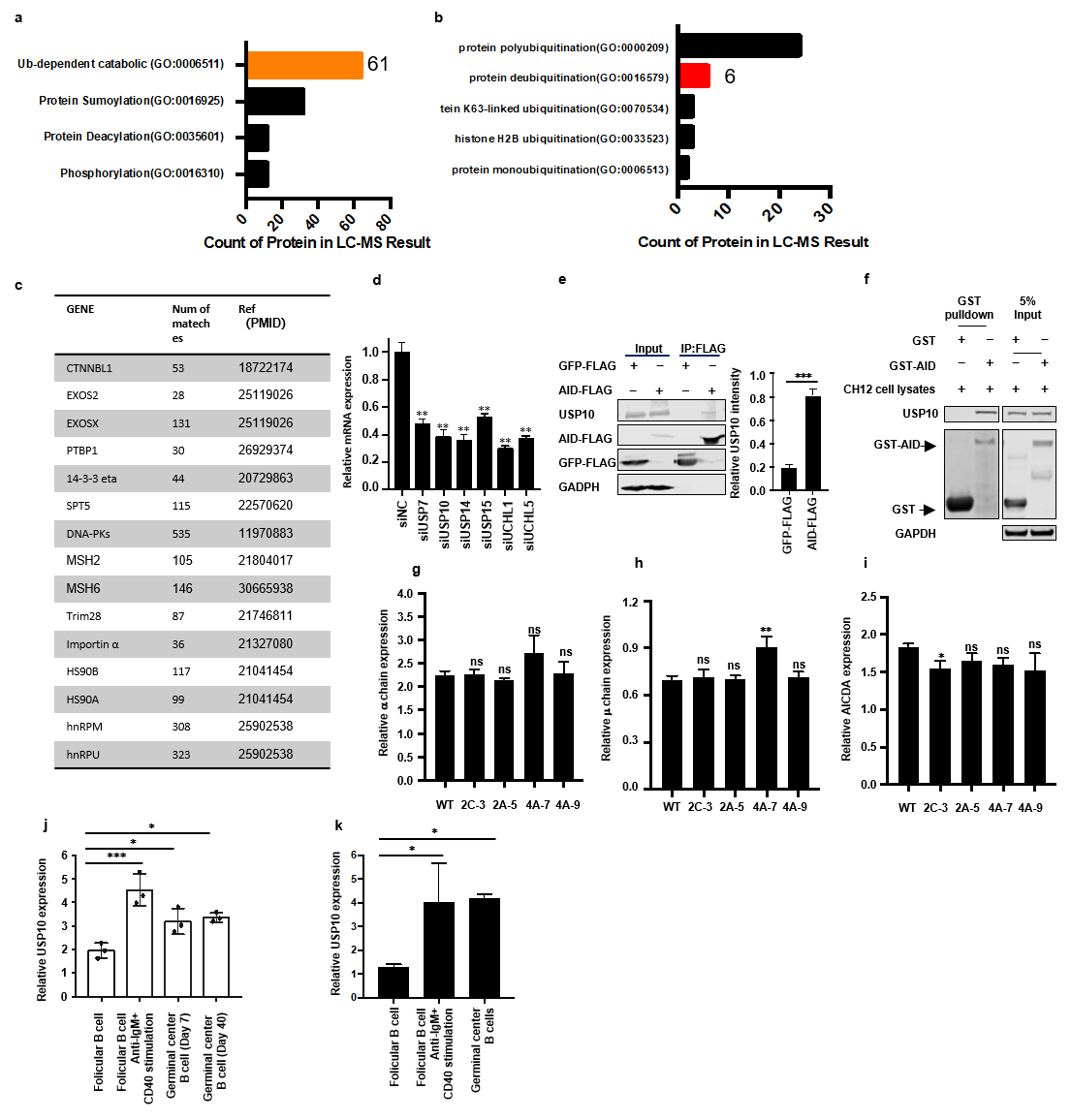


Supplementary Figure 1

**(a)** Sixty-one proteins had functions of Ub-dependent catabolic in 1194 interactors. **(b)** Among the Ub-dependent catabolic associated proteins, there were 6 proteins have functions of deubiquitination. **(c)** The known AID-specific interacting proteins. **(d)** The siUCHL1, siUCHL5, siUSP7, siUSP10, siUSP14, siUSP15 was transfected into 293T cells (Cell density: 6.5×104/cm2) with Lipo2000. The relative mRNA expression was detected by the qPCR assay with primers for related genes. **(e)** USP10 specifically interacted with AID. The 293T cells were transfected with pcDNA3.1-AID-FLAG. Their interaction was examined by CO-IP with anti-FLAG beads and western blotting with anti-USP10 Abs. PcDNA3.1-GFP-FLAG as IP control, GAPDH as was used as a loading control. **(f)** The GST-pull down assay confirmed that USP10 directly interact with AID. GST fusion proteins were prepared following standard protocol. For in vitro binding assays, CH12 cell lysates including USP10 was incubated with GST or GST-AID coupled to GSH-Sepharose. After washing, the bound proteins were separated by SDS-PAGE and immunoblotted with indicated antibodies proteins retained on Sepharose were then blotted with the indicated antibodies.**(g, h and i)** Real-time qPCR analysis of the relative levels of Igμ and Igα germline transcripts (g and h) and Aicda transcription (I) in activated CH12 cells with IL4+anti-CD40+TGF-β according previously described concentration for 48 h, the mice GAPDH mRNA was measured as endogenous controls. **(j and k)** The expression of USP10 in follicular B cells, anti-IgM-anti-CD40 stimulated follicular B cells, germinal center B cells. (j) The USP10 expression in public array data (GSE23925). (k) RT-qPCR determined the USP10 expression in cells.


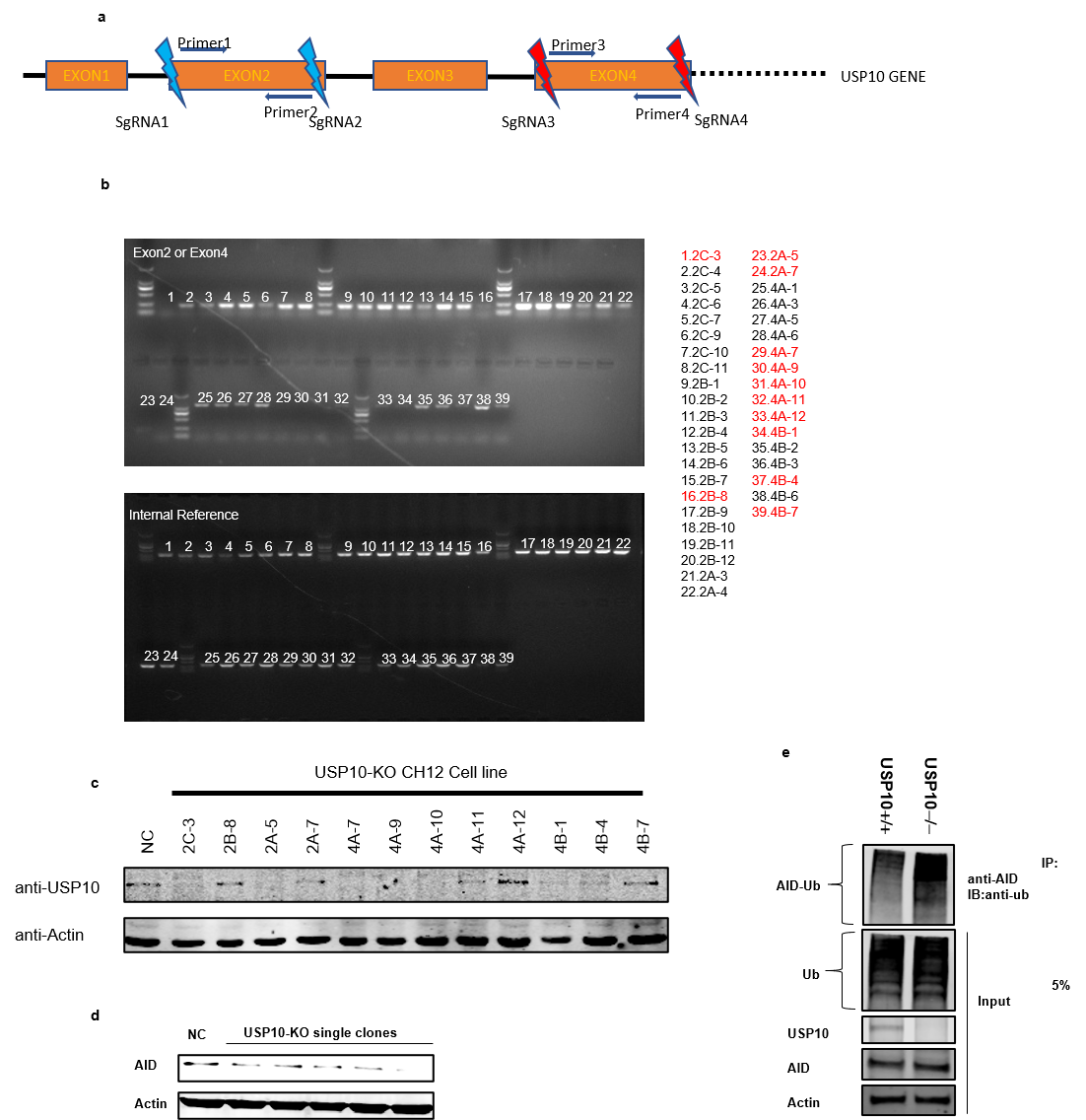


Supplementary Figure 2

**(a)** The design strategy of sgRNA and detecting primers. **(b)** The knockout efficiency of sgRNA was identified by PCR, and the CH12 monoclonal cells with USP10 gene knockout were selected. **(c)** The USP10 knockout monoclonal CH12 cells were identified by western blot. **(d)** The expression level of AID in USP10 knockout CH12 monoclonal cells was detected by western blot. **(e)** The AID ubiquitination in USP10 knockout CH12 cells or wildtype CH12 B cells was detected. The CH12 cells were stimulated with IL4+anti-CD40+TGF-β according to the previously described concentration. The cells were treated with MG132 for 12 h to inhibit AID degradation before harvest. The AID ubiquitination in CH12 cells was analyzed by CO-IP with anti-AID Abs and western blotting with anti-ub Abs. Actin as was used as a loading control.


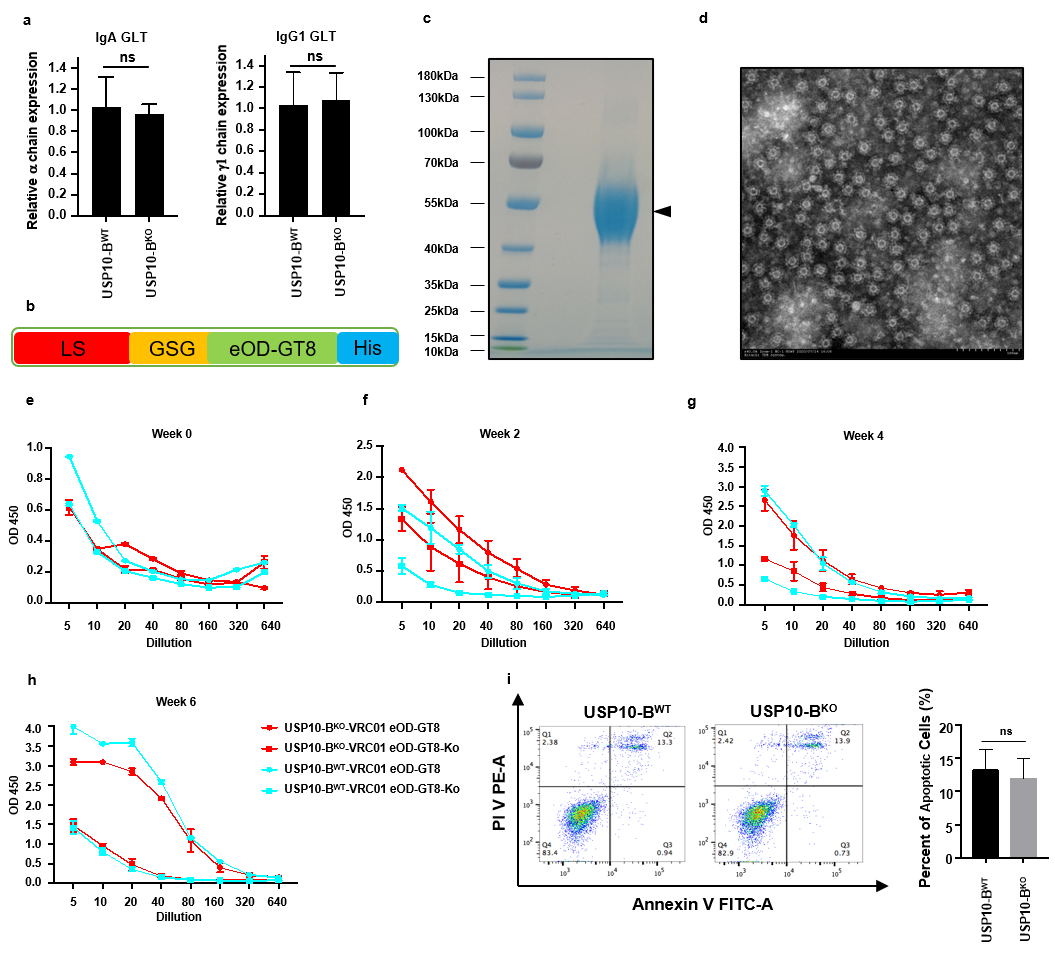


Supplementary Figure 3

**(a)** The expression of GLTs of IgA or IgG1 class-switch B cells in USP10-BWT and USP10-BKO. CD43- naïve splenic B lymphocytes were purified by the magnetic separation (MACS, Miltenyi Biotec). The cells were maintained at 1-3×106 cells/ml in standard culture medium and were treated with 5 ng/ml IL-4 and 25 ug/ml lipopolysaccharide (LPS) so that CSR would be induced to IgG1 or treated with 25 ug/ml LPS, 5 ng/ml TGF-beta and 10 ng/ml anti-IgD for induction of CSR to IgA. The relative GLTs was detected by the qPCR assay with specific primers (PMID:11007474). The experiment data was analyzed using Graphpad Prism. **(b)** The schematic diagram of eOD-GT8 antigen monomer structure. The LS indicated bacterial protein lumazine synthase, CSG is a linker sequence including Glycine Serine Glycine. The eOD-GT8 contains a complete epitope of CD4-binding site that strongly binds to the unmutated form of the VRC01 antibody, His indicated His tag. The eOD-GT8 was displayed on the surface of bacterial protein LS to form a 60 polymer nanoparticle eOD-GT8-60mer. **(c)** The expression of eOD-GT8 was confirmed by Coomassie blue staining. **(d)** The formation of eOD-GT8 60mer nanoparticle vaccine was confirmed by electron microscopy. **(e-h)** The binding affinity of the HIV-1 nanoparticle vaccines including eOD-GT8 or eOD-GT8-KO, which harbors the mutants at D368R and N279A to block the germline VRC01 binding, was detected each two weeks via ELISA assay and plotted as a dilution-course curve. The immunogens (eOD-GT8 60mers) were diluted in PBS (100 μg/ml for 100μl /mouse and mixed at a 1:1 ratio with 100 μl/mouse Alhydrogel 2% (Invivogen)) for at least 20 minutes, and then injected intraperitoneally (i.p) (total volume of 200μl) (n=4). **(i)** The Annexin V and PI double staining assay determined USP10 knockout did not affect the B cells apoptosis. CD43- naïve splenic B lymphocytes were purified by the magnetic separation (MACS, Miltenyi Biotec). The cells were maintained at 1-3×106 cells/ml in standard culture medium and were treated with 5 ng/ml IL-4 and 25 ug/ml lipopolysaccharide (LPS) so that CSR would be induced to IgG1. The cells were then stained by Annexin V-FITC and PI. The percent of apoptocitc cells were determined as Annexin positive cells by FACS.


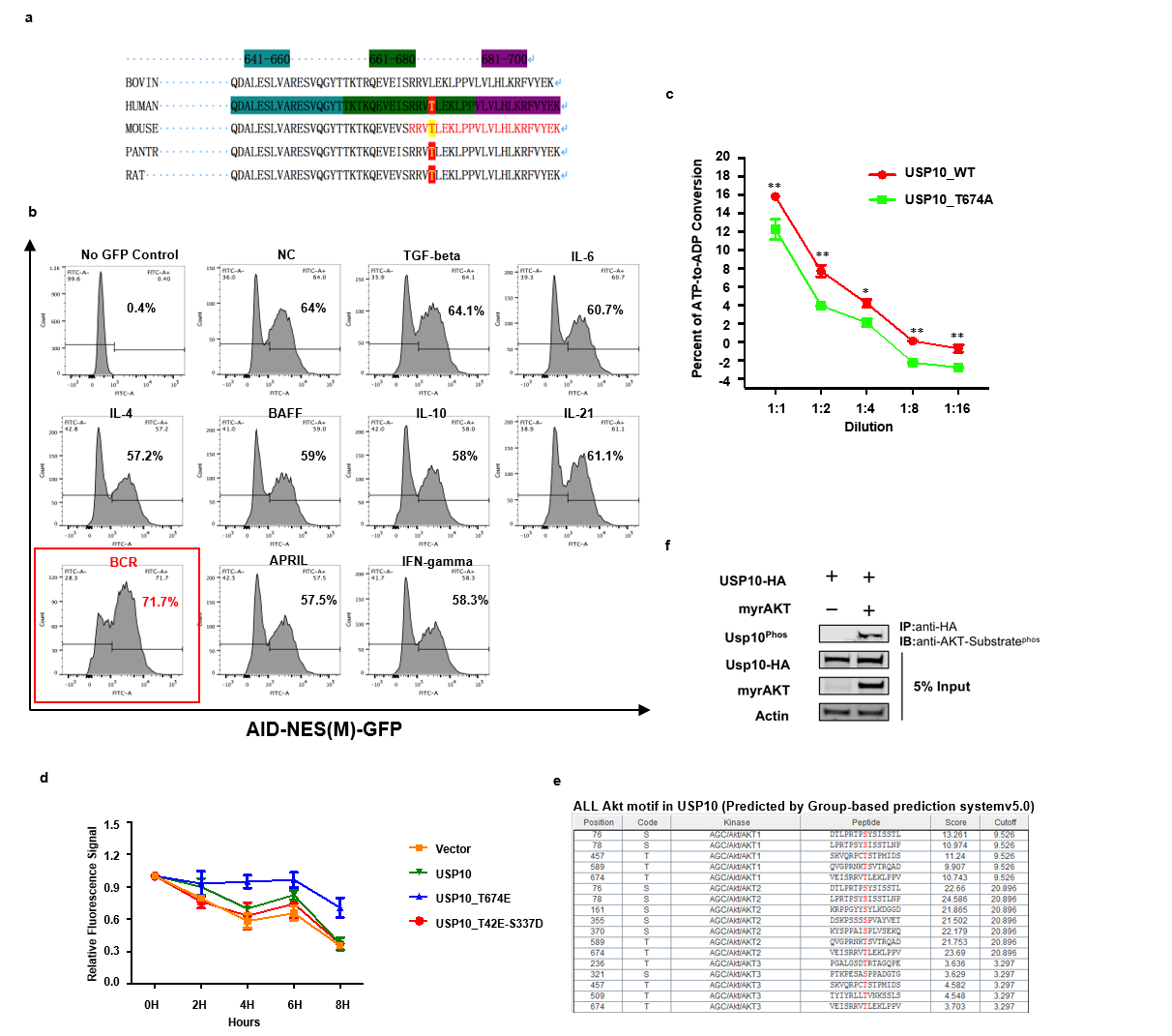


Supplementary Figure 4

**(a)** The amino acid sequence of USP10 nuclear signal and homology among species. **(b)** The Ramos B cells expressing nuclear AID [AID-NES(m)-GFP] were used to screen and the anti-m stimulation of BCR enhanced the nuclear AID-GFP abundance. **(c)** The in vitro kinase assays determined AKT phosphorylates USP10 T674. In vitro phosphorylation was performed using the Promega ADP-Glo kinase assay Kit. In detail, 5 μM wildtype USP10 peptide or T674A USP10 peptide were mixed with 10 μM ATP and 0.25 μg AKT in the phosphorylation reaction system for 20 min. The luminescence absorption of each well was measured by a Promega GLOMAX luminometer reader. The percent of ATP-to-ADP conversion was calculated using a stand curve. (*:*P*<0.05; **:*P*<0.01)**.** (**d)** USP10-T674E overexpression inhibited the nuclear AID-NES(m)-dendta2 degradation but not USP10-T42E-S337D. **(e)** Both Thr42 and Ser337 site in USP10 were assessed not to be of Akt phosphorylation motif via the analysis with Group-based Prediction system 5.0. (F) Phospho-(Ser/Thr) Akt Substrate Antibody confirmed USP10 phosphorylation was directly related to AKT


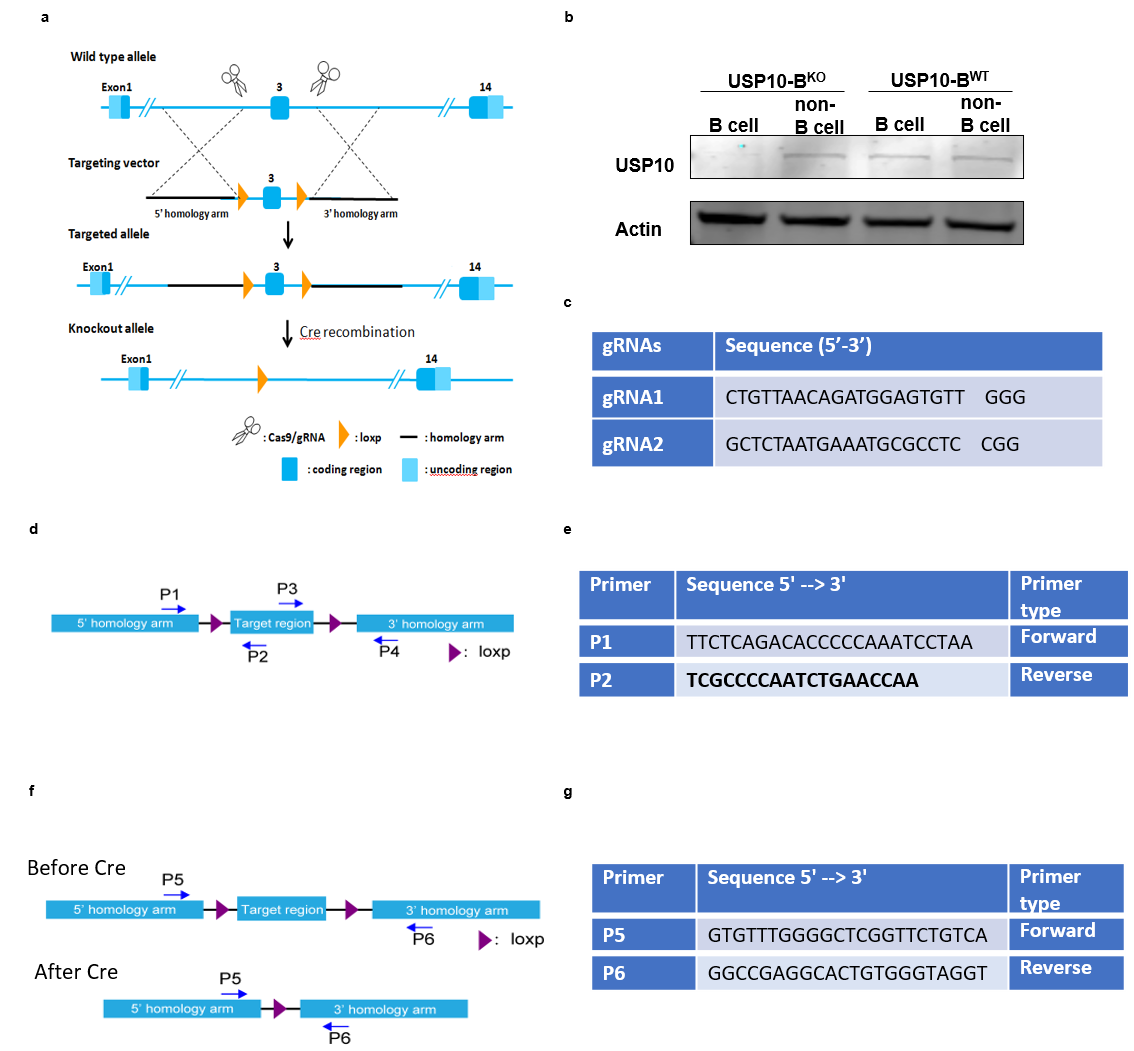


Supplementary Figure 5

**(a)** Construction strategy of USP10 conditional knockout mice. Cas9 mRNA and gRNA were obtained by in vitro transcription. The donor vector was constructed by in-fusion cloning, which contained 3.1KB 5 ‘homologous arm, 0.6KB FLOX region and 3.3KB 3’ homologous arm. Cas9 mRNA, gRNA and Donor vector were microinjected into the fertilized eggs of C57BL/6J mice to obtain F0 generation mice. The positive MICE of F0 generation identified by PCR amplification and sequencing were mated with C57BL/6J mice to obtain 6 positive mice of F1 generation. **(b)** The knockout effect of USP10 in B cells or non-B cells. Mouse spleen B cells were isolated by magnetic beads. USP10 protein expression in B cells and non-B cells was verified by western blot. **(c)** Guide RNA sequence. **(d and e)** Location and sequence of PCR primers used to identify knockout mouse genotypes. **(f and g)** Location and sequence of PCR primers used to confirm Cre shearing.


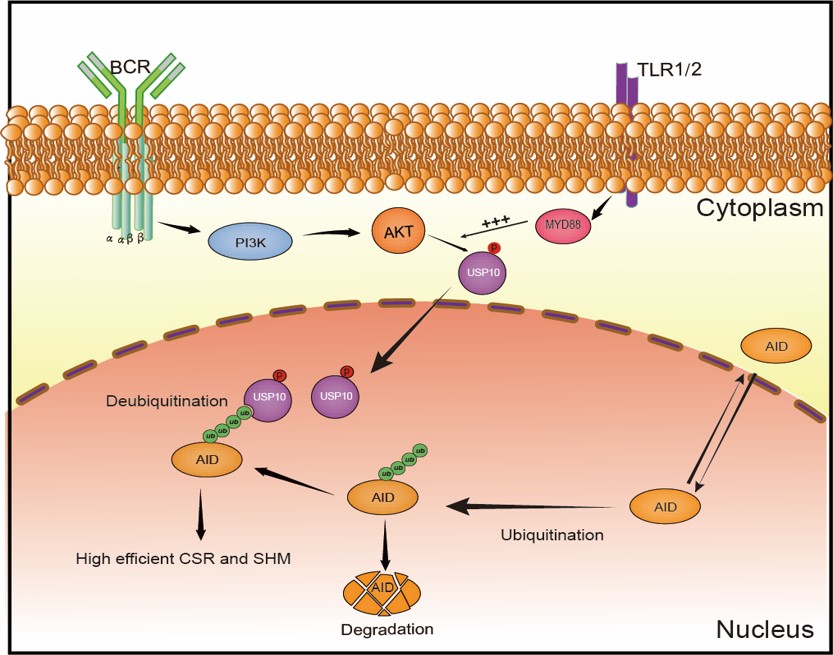


Supplementary Figure 6

**Diagram of this study.** TLR1/2 enhanced the BCR-PI3K-Akt signaling to activate AKT then phosphorylate T674 of USP10. As a result, T674^phos^-USP10 was imported to nucleus and inhibited the degradation of nuclear AID to ensure efficient CSR and SHM.
